# Chemical vapor deposition of 2D materials: A review of modeling, simulation, and machine learning studies

**DOI:** 10.1016/j.isci.2022.103832

**Published:** 2022-01-29

**Authors:** Sayan Bhowmik, Ananth Govind Rajan

**Affiliations:** 1 Department of Chemical Engineering, Indian Institute of Science, Bengaluru, Karnataka 560012, India

**Keywords:** Materials synthesis, Nanomaterials, Theoretical chemistry

## Abstract

Chemical vapor deposition (CVD) is extensively used to produce large-area two-dimensional (2D) materials. Current research is aimed at understanding mechanisms underlying the nucleation and growth of various 2D materials, such as graphene, hexagonal boron nitride (hBN), and transition metal dichalcogenides (e.g., MoS_2_/WSe_2_). Herein, we survey the vast literature regarding modeling and simulation of the CVD growth of 2D materials and their heterostructures. We also focus on newer materials, such as silicene, phosphorene, and borophene. We discuss how density functional theory, kinetic Monte Carlo, and reactive molecular dynamics simulations can shed light on the thermodynamics and kinetics of vapor-phase synthesis. We explain how machine learning can be used to develop insights into growth mechanisms and outcomes, as well as outline the open knowledge gaps in the literature. Our work provides consolidated theoretical insights into the CVD growth of 2D materials and presents opportunities for further understanding and improving such processes

## Graphical abstract

**Figure:**
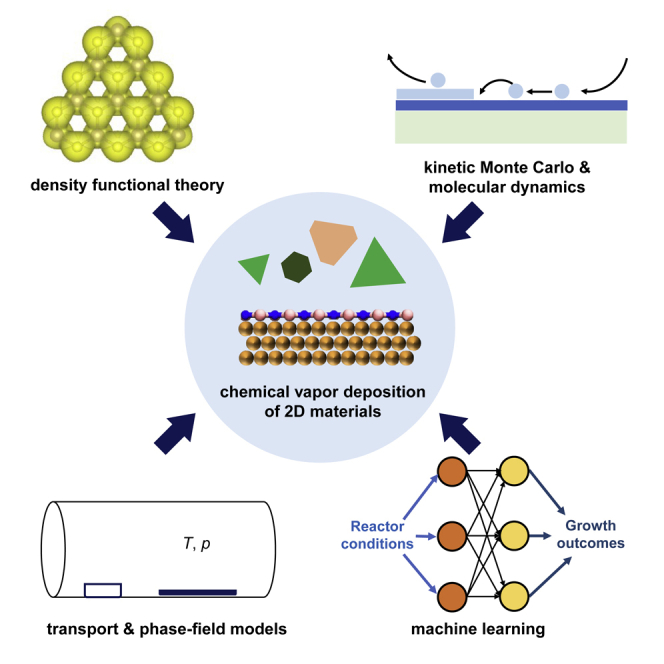

## Introduction

Chemical vapor deposition (CVD) is a scalable route to produce large-area two-dimensional (2D) materials (Cai et al., 2018), required in applications such as optoelectronic devices (Cheng et al., 2019), sensors (Tyagi et al., 2020), and membrane separation modules (Hyun et al., 2019). CVD has been used to grow 2D materials such as graphene (Ani et al., 2018; Muñoz and Gómez-Aleixandre, 2013; Zhang et al., 2013), transition metal dichalcogenides (TMDs) (Jiang et al., 2019; Kumar et al., 2015; Okada et al., 2019; Zhang et al., 2019b), and hexagonal boron nitride (hBN) (Siegel et al., 2018; Stehle et al., 2015; Wang et al., 2019a). Among TMDs, the prominent examples of materials obtained via CVD are molybdenum disulfide (MoS_2_) and tungsten diselenide (WSe_2_). Nevertheless, CVD methods are challenging to reproduce, with variability existing between batches grown in different reactors using the same growth conditions (Narayanan et al., 2019). Further, it is quite difficult to optimize the reaction conditions, such as the temperature, pressure, carrier gas flow rate, and vapor-phase composition required for the synthesis of large-area materials with the desired density of defects. In this regard, modeling and simulation can help in predicting suitable conditions for growth as well as by offering mechanistic insight into the nucleation and growth process. In this review, we discuss the use of tools such as ab initio density functional theory (DFT) (Hohenberg and Kohn, 1964; Kohn and Sham, 1965), kinetic Monte Carlo (KMC) simulations (Gillespie, 1976; Voter, 2007), and reactive molecular dynamics (MD) simulations (van Duin et al., 2001; Senftle et al., 2016) to obtain theoretical insights into the CVD growth of 2D materials.

Quantum-mechanical DFT calculations can be used to predict the thermodynamic and kinetic barriers for the reaction and diffusion processes underlying materials synthesis. However, while DFT calculations allow for the ab initio (first-principles) prediction of potential energy landscapes, they do not involve the simulation of the explicit temporal evolution of the system. In this regard, KMC simulations, based on probabilistic principles, offer the possibility of studying the kinetics of growth over large time scales such as minutes or hours. However, KMC simulations, whether done on a lattice or in an off-lattice manner, only consider a finite number of plausible reaction or diffusion events, for which rates are determined through ab initio calculations or by fitting to experimental data. On the other hand, ab initio MD (AIMD) simulations allow one to use first-principles potential energy surfaces to calculate forces and the resultant atomic velocities, thereby incorporating atomic motion and entropic effects into the model. Because AIMD simulations are computationally very demanding, classical MD simulations using reactive force fields have been explored to simulate chemical reactions, with greatly increased computational speeds, but lower accuracy as compared with AIMD simulations. In this work, we discuss the use of the above-mentioned theoretical techniques to obtain physical and chemical insight into the synthesis of 2D materials.

There have been several review papers focused on the CVD growth of 2D materials from an experimental perspective (Cai et al., 2018; MacKenzie et al., 2010; Muñoz and Gómez-Aleixandre, 2013). From a computational standpoint, Gao et al. and Momeni et al. extensively discussed the simulation tools, including DFT calculations, KMC simulations, and reactive force field MD simulations, that can be used to investigate 2D material synthesis via CVD. While Momeni et al. primarily discussed the theory and basic formulae underlying atomistic to meso/macroscale simulation models in detail, Gao et al. focused more on the growth of specific 2D material systems, particularly graphene (Gao et al., 2018; Momeni et al., 2020). Recently, Dong et al. provided a perspective on the theoretical study of the CVD of graphene and newer 2D materials (Dong et al., 2021). In this work, however, we focus on carrying out an extensive review of modeling, simulation, and machine learning studies aimed at the CVD growth of specific 2D materials, focusing equally on various 2D materials and the knowledge gaps in understanding their CVD synthesis, with the article organized as follows. We first discuss the basic principles of crystal growth and CVD reactors as applied to the synthesis of 2D materials. We dedicate individual sections to modeling the growth of graphene, hBN, TMDs, and newer 2D materials (e.g., silicene, phosphorene, and borophene). In each section, before reviewing and discussing theoretical work, we also summarize the initial experimental work in the area to provide a perspective to the reader as to how the field began. We also review the CVD growth of 2D material heterostructures, which are becoming increasingly popular, as they allow the combination of the favorable properties of various 2D materials. Subsequently, we discuss the use of machine learning strategies to predict and improve CVD processes for 2D materials. We conclude with a consolidated discussion of the knowledge gaps in the field, which could be investigated more deeply in the future.

## Basic principles of CVD and crystal growth

**Figure 1:**
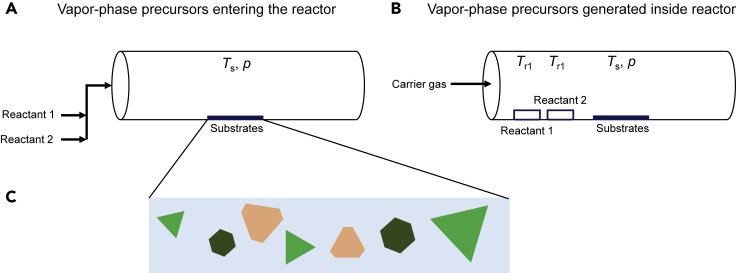
Growth of 2D materials using flow CVD reactors (A) Using gas-phase precursors introduced via mass flow controllers. (B) Using solid-phase precursors sublimated to produce vapors that are transported by a carrier gas and participate in the CVD process. In both cases, growth substrates are placed at an appropriate position inside the reactor oriented horizontally or vertically. Important operating variables include the temperatures, *T*_r1_ and *T*_r2_, of the boats containing the two solid precursors (reactants); the temperature of the substrate, *T*_s_; the pressure in the reactor, *p*; the distance between the boats and the growth substrate*;* and the volumetric flow rate of the carrier gas and reactants. (C) A variety of shapes, including hexagons, triangles, and truncated triangles, of varying sizes, are typically formed on the substrate during the CVD process

CVD reactors used for 2D material synthesis are typically cylindrical in shape, with a diameter of one inch or two inches, and are placed horizontally within a heated furnace. The usual material of construction of the reactor is quartz, so as to sustain the high temperatures to which the system is heated. The furnace is insulated and consists of a single heating zone or three heating zones, with a separate temperature programmer available for each zone. Note that even though the temperatures of the three zones can be set independently, there is heat transfer between the zones such that the final temperatures obtained may be different from the programmed setpoints (Menzel et al., 2012; Sojková et al., 2019; Zheng and Chen, 2017). The growth mechanism depends on the temperature attained inside the reactor, which can be low, moderate, or high, and is determined by heat transfer inside the reactor (Doering and Nishi, 2017). In terms of the pressure used, although atmospheric pressure CVD growth is common (Han et al., 2013; Li et al., 2015b; Vlassiouk et al., 2013), several researchers have investigated low-pressure or vacuum CVD growth of materials (Li et al., 2011; Singhal et al., 2021; Zhang et al., 2019a), with the low pressure maintained using a vacuum pump. To assist with modeling efforts, CVD processes used for 2D materials can be categorized into two types, based on whether the vapor-phase precursors enter the reactor or are generated inside the reactor, with the latter more difficult to model theoretically and control experimentally. In the first type (Figure 1A), gas phase precursors enter the reactor (e.g., methane and hydrogen for graphene; vaporized borazine and nitrogen for hBN; vaporized Mo(CO)_6_ and H_2_S for MoS_2_). In the second type (Figure 1B), solid-phase precursors are placed inside the reactor (e.g., sulfur and molybdenum trioxide or molybdenum pentachloride for MoS_2_), which then sublime and form a vapor phase. Understandably, the second type poses challenges in modeling and simulation, because of the need to account for the kinetics of precursor vaporization/sublimation, and the resulting concentration gradients. Nevertheless, in both types of CVD, a variety of structures are seen on the growth substrate, depending on the temperature, pressure, growth time, precursor concentrations, and flow patterns inside the reactor. Mass flow controllers are used to maintain a constant flow rate of the gases flowing into the reactor (Ciambelli et al., 2011; Hoecker et al., 2016), with the flow rate typically quantified in standard cubic centimeters per minute (sccm), i.e., at conditions of 273.15 K and 1 bar. Thus, modeling studies should take into account the prevalent temperature and pressure conditions to calculate the actual molar flow rate of gases inside the reactor. Ultimately, because many 2D materials have hexagonal lattices, the prominent crystal shapes obtained via CVD are hexagons, triangles, and truncated triangles (Figure 1C). The main types of edges exposed in the synthesized flakes of 2D materials are zigzag (ZZ), armchair (AC), and singly bonded (Klein) edges, with the possibility for unterminated, hydrogen (H) terminated, and hydroxyl (OH) terminated edges. Sometimes, due to severe diffusion limitations on the growth surface, dendritic or fractal structures may also be seen (Chowdhury et al., 2020).

**Figure 2:**
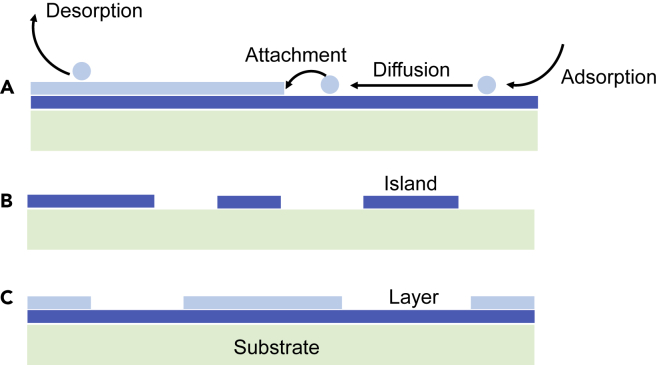
Various modes of growth and the atomistic processes involved in the CVD of a 2D material layer on a suitable substrate (A) “Layer-by-layer” mode (Frank–van der Merwe growth), (B) “isolated islands” mode (Volmer–Weber growth), and (C) “layer-plus-islands” mode (Stranski–Krastanov growth). Steps involved in the CVD process, such as adsorption, diffusion, attachment, and desorption of adatoms are depicted in panel (A)

The CVD synthesis of a material involves several competing processes, such as vapor-phase transport of reactants, adsorption and desorption of precursors on the surface, nucleation and growth of the crystal involving attachment and detachment of adatoms, and the diffusion of adatoms on the surface (Zhang and Lagally, 1997). These processes are depicted in Figure 2A. Furthermore, there are three main modes for epitaxial crystal growth (Markov, 1995). These are the “layer-by-layer” mode (also called Frank–Van der Merwe growth, depicted in Figure 2A), the “isolated islands” mode (also called Volmer–Weber growth, depicted in Figure 2B), and the “layer-plus-islands” mode (also called Stranski–Krastanov growth, depicted in Figure 2C). For the growth of monocrystalline large-area 2D materials, the layer-by-layer mode is the most preferred followed by the layer-plus-islands mode, as opposed to the isolated islands mode, because the latter involves small flakes growing in a disconnected manner. For obtaining polycrystalline 2D materials, any of the above modes of growth may be sufficient. The mode of growth depends upon the free energetics and kinetics of the various processes mentioned above and is an important prediction that theory can be used to make. Indeed, if the lateral growth of the 2D material is faster than the nucleation of another layer, then layer-by-layer growth will take place; otherwise, there will be a tendency for island-type growth to occur.

## Modeling of graphene CVD growth

### Experimental background

Graphene, the first 2D material to be isolated, has found use in several application areas, such as separation processes (e.g., water desalination (Song et al., 2018) and gas separations (Yoo et al., 2017)), electronics (Avouris and Xia, 2012; Wang et al., 2012), energy storage (Olabi et al., 2021; Raccichini et al., 2014), and biosensing (Chung et al., 2013). While graphene was initially isolated using scotch-tape exfoliation (Novoselov et al., 2004), this method is not scalable and leads to small sizes and quantities of 2D flakes. Thus, researchers turned to other techniques for producing graphene: liquid-phase exfoliation (Nicolosi et al., 2013), a top-down approach, and CVD, a bottom-up approach. However, graphene obtained via liquid-phase exfoliation is also limited in its lateral size and has several defects due to the vigorous conditions presented by ultrasonication (Skaltsas et al., 2013). To overcome these challenges, over the years, researchers have perfected CVD as a potential growth method for graphene. Few-layered graphene was first synthesized via CVD in 2007 (Obraztsov et al., 2007). Subsequently, single-layered graphene was synthesized via CVD by three different research groups in 2008-10 (Cao et al., 2010; Kim et al., 2009; Reina et al., 2008). The growth of graphene involves the use of methane and hydrogen gases as reactants. Several experimental and theoretical studies tried to understand the mechanism of graphene nucleation and growth on various substrates. Tetlow et al. reviewed progress in this area extensively (Tetlow et al., 2014).

**Figure 3:**
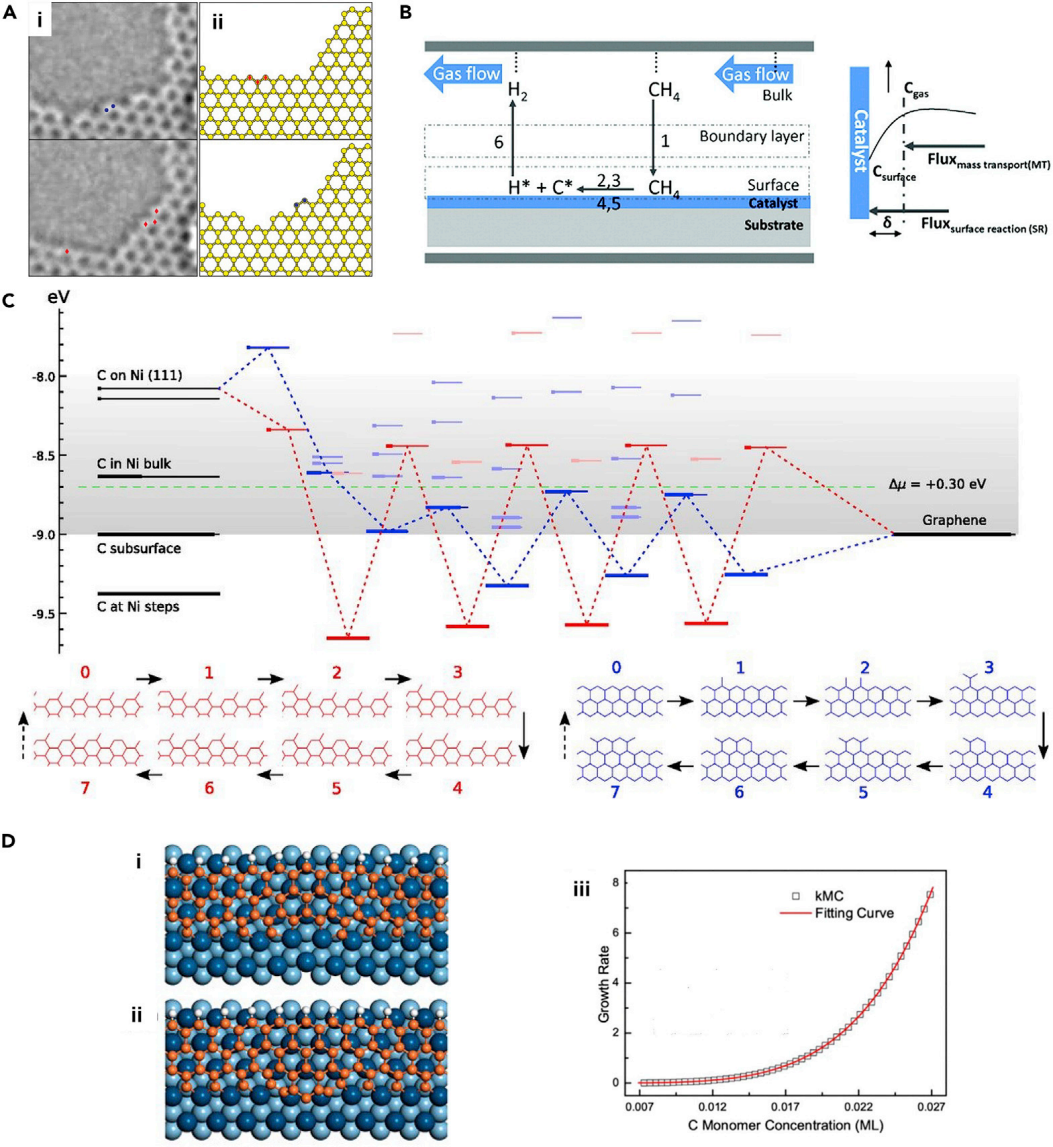
Modeling and experimental studies focused on understanding the CVD synthesis of graphene (A) Reconfiguration of the graphene edge (i) TEM image depicting the conversion of an edge in graphene from an armchair configuration (top) to a zigzag configuration (bottom) (ii) Conversion of a zigzag edge (in red) to an armchair edge (in blue) as seen in KMC simulations. Adapted with permission from Girit et al. (2009). Copyright © 2009, The American Association for the Advancement of Science. (B) Schematic of transport phenomena inside a graphene CVD reactor. Adapted with permission from Bhaviripudi et al. (2010). Copyright © 2010, American Chemical Society. (C) Energetics of the A5’ (red) and zigzag (blue) edge growth on Ni substrate. The carbon atoms fill the energy levels, which represent the energetics of the n^th^ carbon atom attaching to a row containing (n-1) atoms, as they transform from source (leftmost levels, substrate) to products (rightmost levels, graphene). The chemical potential of carbon atoms as determined by the feed gas is represented by the green line and the stable configurations of growth are depicted below. Adapted with permission from Artyukhov et al. (2012). Copyright © 2012, National Academy of Sciences. (D) Graphene growth on Ir(111). (i) Carbon attached at edge sites. (ii) C_5_ clusters attached at edge sites. (iii) Variation of graphene growth rate with carbon monomer concentration. Adapted with permission from Qiu et al. (2018a). Copyright © 2018, American Chemical Society

In terms of experimental studies focused on understanding the CVD process, Girit et al. examined the mechanism of edge reconstruction in graphene via aberration-corrected transmission electron microscopy (AC-TEM), as shown in Figure 3A(i). A movie generated from the imaging of the edges in graphene established the stability of the zigzag edge. The authors also investigated whether a KMC model could predict the experimentally observed results (Figure 3A(ii)). Although the atomic migration probabilities were calculated from an Arrhenius dependence on the energy barrier obtained from ab initio calculations, the ejection and addition probabilities were set to match experimental results, thus leaving room for future improvement where all rates could be calculated from first principles. The dynamics of the edges and holes were predicted to be similar to the observed results (Girit et al., 2009).

Subsequently, Bhaviripudi et al. experimentally investigated the role of the pressure in the reactor in the kinetics of the CVD synthesis of graphene using copper (Cu) as a catalyst. Schematics of the proposed mechanism and the concentration gradients in the system are illustrated in Figure 3B (Bhaviripudi et al., 2010), depicting the vapor-phase boundary layer, which separates the growth substrate from the bulk gas. The authors demonstrated the crucial role of kinetics in forming uniform graphene sheets of large area. The thermodynamics of the system remained unaffected, but the kinetics of the CVD growth changed based on whether low pressure, atmospheric pressure, or ultra-high vacuum conditions were used. The researchers found out that growth under atmospheric pressure and at higher concentrations of methane is not self-limiting in comparison to the observations in the low-pressure growth case (Bhaviripudi et al., 2010). The researchers also developed a reliable kinetic model, which was applicable at low (10 mtorr) and very low pressures (mtorr), for a broad range of hydrocarbon precursor gases including a mixture of methane (CH_4_), ethylene (C_2_H_4_), and hydrogen (H_2_) gases. The results obtained were validated by their experiments and the “dual role” of hydrogen in graphene generation using Cu catalyst was explained. In other words, a threshold partial pressure of hydrogen is necessary to start the nucleation process. However, hydrogen is a known etchant, both in gaseous and adsorbed form. Thus, an optimal concentration of hydrogen is essential for supporting graphene growth. This dual role of hydrogen also affects CVD via the system temperature. Indeed, etching reactions are more probable at higher temperatures (Mehdipour and Ostrikov, 2012), consistent with an increased tendency to surmount the thermal barrier for C-C bond breaking. In another work, Kim et al. investigated the energetics of graphene growth and nucleation from Arrhenius plots of the growth rate versus the inverse of the system temperature, as observed in CVD experiments on a Cu substrate. Their analysis resulted in a model which could explain the synthesis of single-crystalline graphene sheets (Kim et al., 2012). Other experimental work focused on the controlled synthesis of graphene having tunable edges (Ma et al., 2013). Safron and Arnold studied the atmospheric pressure-driven CVD of graphene on Cu from methane. The role of the critical methane concentration (CMC) was demonstrated in this work. The authors showed that the nucleation and growth of graphene occurred above the CMC. Instead, at the CMC, the attachment and detachment rates of the intermediates were roughly the same. Furthermore, etching was observed at a methane concentration below the CMC. The reaction intermediates and the mechanisms were elucidated in this work using experimental data obtained from field-emission scanning electron microscopy (SEM) and Raman spectroscopy of the graphene samples. The authors postulated a model to predict the kinetics of nucleation and growth, as well as to identify the building units in the mechanism (Safron and Arnold, 2013). Knowledge of the building units is crucial in the development of chemical reaction mechanisms and simulation models for CVD synthesis, and thus more experimental efforts can be made in this direction in the future. There has also been experimental work focused on other graphene geometries, such as nanoribbons. Jacobberger et al. introduced a synthesis method for graphene nanoribbons via CVD. The study was performed on Ge substrates and paved the way for possible applications of nanoribbons in electronic circuits (Jacobberger et al., 2015).

### Progress and prospects on the theoretical front

On the modeling front, the first theoretical studies focused on the vapor-phase synthesis of graphene came in the year 2012. Artyukov and Yakobson carried out detailed first-principles calculations of the growth rate of graphene edges using a step-flow model of crystal growth (Artyukhov et al., 2012). The authors modeled the migration of carbon atoms from the feed to the nickel (Ni) catalyst surface and their attachment to the growing lattice. Subsequently, they calculated the energies of the various intermediate states to elucidate the sequence of steps involved in the graphene growth process (Figure 3C). The atoms were observed to attach at the edges and not form many defects in the presence of substrates. This work was successful in explaining the anisotropy of the growth process, the kinetics of growth, and the morphology of the islands, thereby providing physical insight into previous experimental results, including the synthesis of carbon nanotubes (Artyukhov et al., 2012). Meng et al. studied the kinetics of growth of carbon structures on Ni(111) at various temperatures using classical reactive MD simulations by employing the ReaxFF potential (Meng et al., 2012). The study found that higher concentrations of carbon atoms favor graphene island formation and lower concentrations yield dissolution of carbon into the Ni surface. Thus, extended sp^2^ networks are not feasible at a low carbon concentration (due to dissolution of growth precursors) and at a high concentration (due to the formation graphene islands), thereby suggesting an optimal carbon concentration for large-area growth. The authors further reported that graphene islands can act as nucleation centers and allow growth by the addition of carbon (Meng et al., 2012). The Ding group found out that the orientation of graphene islands is determined by the interactions operating between the graphene edge and the catalyst surface (Zhang et al., 2012). This important finding provided an explanation for various experimental observations, such as the crossing of grain boundaries (formed by adjacent facets of polycrystalline catalyst surfaces) by monocrystalline graphene; synthesis of graphene with a domain size larger than the domains in the catalyst surface; and correlation between the graphene flake orientation and the catalyst surface (Zhang et al., 2012).

In other work, researchers investigated the mechanism of nonlinear graphene growth on metal surfaces using ab initio calculations and KMC simulations. Particularly, Wu et al. modeled the addition of smaller carbon species along with adsorption and diffusion phenomena (Wu et al., 2012). The authors elucidated the mechanism of nucleation and growth by studying the step sites on the substrate and carbon attachment to the edges, respectively. The lattice mismatch between graphene and the substrate resulted in inhomogeneous growth, an aspect which deserves more attention in future work, as it can explain different growth regimes in the same CVD reactor. The authors could not investigate perpendicular growth of carbon chains larger than seven due to the finite substrate size considered. Future studies can focus on the growth of larger carbon chains by using larger system sizes. Nevertheless, the methodology to investigate simultaneously adsorption, diffusion, and growth developed in their paper can be extended to understand the growth of other heteroepitaxial structures (Wu et al., 2012). Theoretical studies have also been carried out using semi-empirical electronic methods. For example, using a tight-binding model, semi-empirical molecular orbital theory, and reactive empirical bond order (REBO) potential-based calculations, Elliot et al. showed that graphene is thermodynamically stable on crystalline Ni(111). The researchers considered the “complementary” crystallization of graphene and metal in their calculations. Thereby, they demonstrated a crystallization process involving the simultaneous heterogeneous nucleation of graphene and metal, which could provide better control of the growth process for both graphene and carbon nanotubes (Elliott et al., 2013).

Another work investigated methane dissociation on the Ni (111) surface with the help of AIMD simulations (Shibuta et al., 2013). The study found that methane dissociates into carbon and hydrogen atoms via the chemisorbed CH_3_, CH_2_, and CH intermediates on the Ni surface. The authors proposed that carbon atoms diffuse into the subsurface resulting in the precipitation growth of graphene on the catalyst surface (Shibuta et al., 2013). Monolayer graphene was formed in the temperature range between 1100 and 1200 K. The growth of graphene on other nickel surfaces, e.g., Ni(110) and Ni(100), was investigated by Mafra et al. (Mafra et al., 2018). The authors observed that multilayer growth was possible only using Ni(100). No carbon structures were observed at lower temperatures (below 800°C) due to reduction in the reactivity rates of methane. Nevertheless, a higher growth time can be employed to overcome the reduced reactivity. Hence, a tradeoff between the growth time and temperature is evident in the monolayer growth of graphene on Ni substrates, an aspect that can prove useful for the diagnosis of reasons for failed growth. The authors further proposed that the planar structure is the most stable one with half of the carbon atoms attached to the “on top” position and the other half to the “hollow hcp” position. However, there are disagreements on the most stable structure of graphene on Ni(111) and deeper investigations are needed to address the problem (Mafra et al., 2018). Regarding growth on other surfaces, Page et al. investigated the nucleation of graphene on Ni, Cu, and iron (Fe) surfaces (Page et al., 2013). The authors found that weakly binding catalysts such as Ni and Cu are better for graphene growth as they do not degrade with time. Nevertheless, Ni and Cu require a higher carbon concentration on the surface for graphene growth to take place. On the other hand, although quantum-chemical calculations showed that Fe facilitated graphene precursor formation even at lower carbon densities, Fe(111) suffers degradation due to a strong interaction with carbon. The authors postulated that graphene growth, like carbon-nanotube growth, demonstrates a substantial correlation with the catalyst-carbon interaction (Page et al., 2013).

Apart from temperature and growth time, the flow profile of vapor inside the reactor can play a critical role in modulating CVD growth. Li et al. studied the dynamics of the gas-phase in growth of graphene on Cu using computational fluid dynamics (CFD) (Li et al., 2015a). The authors calculated the deposition rate of carbon on the surface during the growth of graphene in a horizontal tube furnace. The researchers considered 3D, compressible flow in the laminar regime along with chemical reaction at the surface, wherein the flow is governed by the continuum continuity, Navier-Stokes, and heat equations. The surface deposition rate was found to depend on the surface reaction rate constant and the mass transfer coefficient of methane, both of which are affected by pressure. Indeed, under low pressure conditions, the deposition rate increased exponentially with temperature whereas the increase was moderate under atmospheric conditions. The deposition rate was shown to depend on the placement of the Cu substrate. Accordingly, a higher concentration of unsaturated species in the downstream section resulted in thicker growth of graphene. This study is important as it provides insights into the transport phenomena occurring during graphene growth, where under lower pressures, the surface reaction process is controlling but at atmospheric pressure, the mass transport process is controlling (Li et al., 2015a). In another comprehensive work, Zhang et al. discovered the role of hydrogen in terminating the graphene edges (Zhang et al., 2014). They found out that at low hydrogen pressures, the graphene edges tend to attach to the surface of the catalyst as the edges are not terminated by the hydrogen atom. This prohibits the diffusion of carbon species to the bottom and favors growth of single layers of graphene. At higher pressures, the graphene edges were passivated by the hydrogen atoms and hence the formation of an adlayer beneath the top layer was favored. This observation paves the way for controlled synthesis of bilayer graphene or few-layered graphene. Further, Li et al. also observed in their KMC simulations that graphene edges are H-passivated at higher hydrogen partial pressures and metal passivated at lower pressures. With the help of DFT calculations, the researchers showed that growth at the hydrogen-passivated edge can proceed via C_2_ or CH attachment as both produce sp^2^ structures (Li et al., 2017). CH attachment is generally not favorable at the edges due to the former’s lower desorption barrier, whereas, C_2_ attachment is easier due to a higher desorption barrier at the edges. However, at higher H_2_ partial pressures, the CH concentration is higher than the C_2_ concentration and attachment at the H-passivated edge is driven by the CH species (Li et al., 2017). Thus, the growth mechanism not only depends on reaction energetics, but also on species concentrations. This coupled effect should be investigated in more detail in the future.

Research work focused on evaluating the accuracy of theoretical methods is also required to ensure that the predictions made using simulation tools are reliable. Shu et al. studied the various carbon species that form during the CVD growth of graphene using first-principles methods. They found that the CH and C monomers are predominant on the Cu surface; and on Ni, C monomers were larger in number during graphene growth. On Ir and Rh surfaces, C species were stable and drove the CVD growth process (Shu et al., 2015). Note that a strong binding between the transition metal and carbon species is required for the latter to get attached to the surface and contribute to the nucleation and growth of graphene. The binding energies of the carbon species to the surface decrease on addition of hydrogen. To this end, dispersion corrections to DFT must be added to accurately obtain the binding energy and incorporate the weak van der Waal’s interaction between the carbon species and the substrate. Shu et al. reported that the binding energies of the active species, CH_*i*_ (*i* = 0,1,2,3) are dependent on the method (type of functionals and dispersion correction), an aspect which should be investigated more deeply in the future. In fact, the stabilities of the various species also depend on the temperature and the type of substrate surface. An increase in the temperature causes the reduction of chemical potential of hydrogen and leads to further dehydrogenation of the species (Shu et al., 2015). Simulation studies taking into account the effect of temperature on growth are essential to make progress in this area. In DFT calculations, thermal effects are mainly incorporated via the harmonic oscillator approximation. In this regard, Andersen et al. found that the harmonic oscillator representation may not be accurate at higher temperatures on both solid and liquid Cu substrates (Andersen et al., 2019). Hence, the researchers considered a hindered translator/rotator (HTR) and 2D ideal gas model for the adsorbate. It was shown that in the absence of diffusional and rotational barriers, the HTR model reduces to the 2D ideal gas model. Observing the Gibbs free energy of formation of hydrocarbon species, the authors concluded that the species containing lesser number of hydrogen atoms were more stable at higher temperatures for CVD growth on solid Cu. The authors observed that for a decrease in methane to hydrogen partial pressure ratio (at fixed total pressure) or an increase in total pressure, the hydrogenated species become more stable in agreement with previous studies. However, the authors reported in disagreement with Zhang et al. (2014) that an increase in the methane-hydrogen partial pressure ratio leads to multilayer growth of graphene, a mismatch that may be attributed to the lack of thermal corrections to the internal energy and entropy of the adsorbents in previous investigations. Most of the previous studies considered a harmonic oscillator framework, which may not be applicable in case of low rotational or diffusion barriers. Andersen et al. further observed a lower stability for all the hydrocarbon species in the case of CVD growth on liquid Cu. The carbon dimer is expected to have a higher coverage than other carbon clusters owing to its presence on a local minimum on the free energy surface. Therefore, the dimer forms a candidate species to propagate growth after nucleation as observed in KMC simulations. The authors postulated based on stability arguments that both hydrogen-terminated and metal-terminated edges are possible during CVD growth of graphene on Cu substrates, which contradicts previous studies where researchers advocated for the presence of hydrogen-passivated edges at higher hydrogen partial pressures. Such contradictions call for further research in this area to firmly investigate and establish a mechanism of growth, while considering the stability of various edge terminating groups (Andersen et al., 2019).

Interesting physical observations regarding the graphene growth process have also been reported. Artyukhov et al. inferred that the symmetry of the carbon islands formed is not strictly related to the symmetry of graphene (Artyukhov et al., 2015). Using DFT calculations, they found that the symmetry of stacking on the surface is lower than that of either the hexagonal graphene or the catalyst surface. The study found out that symmetry breaking is evident at the edges, resulting in various stable structures (Klein or zigzag) depending on the relative orientation of the graphene flake and the substrate. This phenomenon results in equilibrium shapes which violate the inversion symmetry of graphene mildly. The symmetry-lowering interactions were observed to amplify the growth kinetics exponentially, leading to the rapid growth of Klein edges rather than zigzag edges (Artyukhov et al., 2015). Subsequently Jiang and Hou developed a KMC scheme augmented with DFT calculations to model the growth of graphene on arbitrary catalyst surfaces with lattice mismatch. They proposed a growth mechanism based on the various active sites available on the catalyst surface for graphene growth. The authors found that the rate-determining step for graphene growth was different for various growth fronts, thereby indicating it to be a geometric effect (Jiang and Hou, 2015). In this regard, accurate information on the energetics of atomic-scale transformations in graphene is crucial for the development of reliable theoretical models. Accordingly, Skowron et al. reviewed the effect of defects on the physical and chemical properties of graphene. The study revealed that there were various knowledge gaps, in the areas of energetics of formation and migration of vacancy defects, realignment of bonds, and displacements of crystallographic planes (Skowron et al., 2015). Göltl et al. developed a KMC model to understand the growth of graphene nanoribbons on Ge(001). By considering aspects such as precursor formation, row nucleation, and row growth, the authors were able to reproduce experimental observations in their simulations, concluding that an anisotropic stabilization of growth precursors favored ribbon formation (Göltl et al., 2020). Future work could explore the formation of nanoribbons of graphene and other 2D materials on various substrates, and strategies to control their width, from a computational standpoint. In another work, researchers verified that first-principles calculations coupled with KMC simulations can accurately predict graphene growth kinetics on Ir(111), thereby enabling a deeper understanding of growth mechanisms and the development of better synthesis routes. The carbon monomers were observed to be present in large numbers on Ir(111) and thus, *monomers* attached to all the sites that were exothermic with respect to carbon attachment (Figure 3D(i)). On endothermic sites, growth occurred via exothermic *cluster* attachment (Figure 3D(ii)). Thus, the growth rate is a combination of the attachment kinetics of monomers and large clusters. The variation of growth rates with carbon monomer concentration is shown in Figure 3D(iii) (Qiu et al., 2018b). Recently, Zhang and Duin investigated the CVD growth of graphene on silicon carbide using classical reactive MD simulations. This work involved the development of a silicon/hydrogen/graphene force field, using which the operating temperatures were found to be optimal between 1000 and 3000 K (Zhang and Duin, 2020). Future work can explore the use of ReaxFF models to understand the controlled growth of graphene on various substrates.

To conclude this section, several theoretical studies, based on various simulation methods, have provided critical insights into the nucleation and growth of graphene, as reviewed above and summarized in Table 1. Nevertheless, there is a considerable lack of studies which probe the detailed chemical reaction mechanism of graphene nucleation and growth, while considering edge functionalization, and use it to predict the kinetics of CVD synthesis of graphene. The inclusion of the effects of temperature, pressure, precursor concentration, flow profile, and substrate placement will impart further realism to the modeling efforts. Indeed, in Table 1, one clearly sees a lack of studies focused on nucleation and transport modeling. Such investigations would allow researchers to predict optimal conditions for CVD growth on various substrates, based on the atomic-scale chemistry of the process. The pursuit of such research directions may be a promising endeavor in the future.

**Table 1:** Summary of the studies addressing different focus areas in graphene CVD growth using various theoretical methods

Focus Area ↓	Method →				
	DFT	MD	KMC	AIMD	CFD/Phase-Field Modeling
Nucleation	Wu et al. (2012)	Meng et al. (2012), Elliott et al. (2013)			
Growth	Artyukhov et al. (2012), Wu et al. (2012), Artyukhov et al. (2015), Jiang and Hou (2015), Qiu et al. (2018b)	Meng et al. (2012), Elliott et al. (2013), Zhang and Duin (2020)	Wu et al. (2012), Jiang and Hou (2015), Göltl et al. (2020), Qiu et al. (2018b)		Momeni et al. (2021), Li et al. (2015a)
Role of the Substrate	Zhang et al. (2012), Mafra et al. (2018), Shu et al. (2015), Zhang et al. (2014), Jiang and Hou (2015)			Shibuta et al. (2013), Page et al. (2013), Jiang and Hou (2015)	
Transport Modeling					Momeni et al. (2021), G. Li et al. (2015a)

## Modeling of hBN CVD growth

### Experimental background

Hexagonal boron nitride (hBN), also known as white graphene, is an insulating 2D material that has found application in several areas, such as optoelectronic devices (Caldwell et al., 2019; Cheng et al., 2019; Ougazzaden et al., 2021), molecular/ionic separations (Gao et al., 2017; Lei et al., 2013), and osmotic power harvesting (Kim et al., 2019; Macha et al., 2019; Yazda et al., 2021). An early work by Allendorf and group investigated the kinetics of the gas-phase CVD growth of boron nitride (BN) from BCl_3_ and NH_3_ precursors. The study found the presence of Cl_2_BNH_2_, which occurs during the reaction of BCl_3_ and NH_3_, to be a critical precursor in the CVD growth of BN (Allendorf et al., 1995). Later, in 2010, few-layered and single-layered hBN were first synthesized using CVD by Kong and coworkers using borazine (B_3_N_3_H_6_), aided by nitrogen as the carrier gas at a temperature of about 400°C on a Ni surface (Shi et al., 2010). In other work, Stehle et al. studied the growth of hBN crystals and showed that they change shape depending on the distance between the substrate (Cu) and the precursor. They found out that the heating method of the precursor influences the morphology of the hBN films (Stehle et al., 2015). Song et al. studied the thermokinetics of the CVD growth of hBN from solid ammonia borane. The optimal carrier gas flow rate, temperature of growth and heating, and growth time were determined. The researchers were able to produce large hBN film domains of 80 μm^2^ having 95–100% coverage ratio, thereby indicating the important role played by the aforementioned process parameters in the CVD process (Song et al., 2016). In a review article, McLean et al. observed that the CVD growth of BN is not very well understood due to the vast variability in precursors, reaction conditions, and catalysts. Hence, there is adequate scope for theoretical and experimental investigations that might reveal deeper insights into the mechanism of crystal formation, reaction pathways, and related understanding of the atomistic processes at play (McLean et al., 2017).

### Progress and prospects on the theoretical front

**Figure 4:**
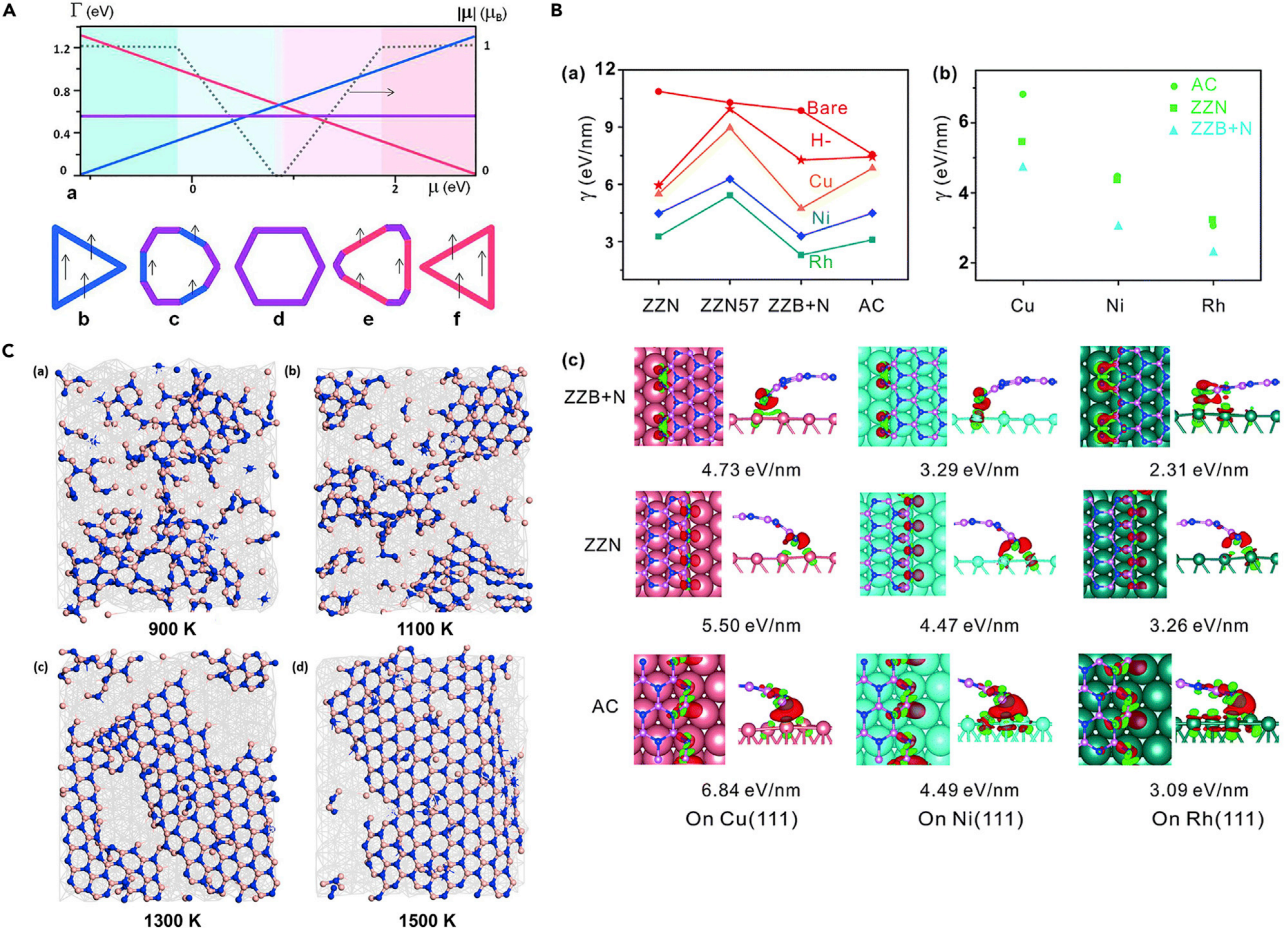
Modeling studies focused on understanding the CVD synthesis of hBN (A) Energetics of hBN edges. (a) Energies of nitrogen-rich zigzag (blue), boron-rich zigzag (red), and armchair (purple) edges plotted against the chemical potential of boron. The black dotted line represents magnetism per unit perimeter, |μ|, and changes with the consideration of various equilibrium hBN shapes as depicted in (b-f). The outline colors are the same as that used in panel (a).Adapted with permission from Liu et al. (2011a). Copyright © 2011, American Chemical Society. (B) Formation energies of various edges. (a) Bare and H-terminated unsupported hBN, as well as hBN on Cu (111), Ni (111), and Rh (111). (b) Armchair (AC) edge, nitrogen-rich zigzag (ZZN) edge, and the Klein edge derived from the boron-rich zigzag edge, with dangling nitrogen atoms (ZZB + N), on the (111) surfaces of Cu, Ni, and Rh. (c) Charge difference distribution profiles between BN nanoribbons and various substrates. Loss of charge is represented by green color and gain of charge is represented by red color. Reproduced from Zhao et al. (2015) with permission.Copyright © 2015, Royal Society of Chemistry. (C) Formation of hBN sheets on Ni as observed in reactive MD simulations at (a) 900 K, (b) 1100 K, (c) 1300 K, and (d) 1500 K. Adapted with permission from Liu et al. (2017). Copyright © 2017, American Chemical Society

On the theoretical front, the edge energies of BN were first calculated by Yakobson and coworkers. The authors considered both N-rich and B-rich zigzag edges and reported the variation of the edge energies as a function of the chemical potentials of B and N (Figure 4A(a)), thus offering insights on how to control the morphology of hBN flakes (Figure 4A(b-f)) and determine the resultant electronic and magnetic properties of BN (Liu et al., 2011a). The authors developed a methodology to describe the energetics of the graphene edge in any arbitrary crystallographic direction at a particular chemical potential. The Wulff construction method was applied to determine the equilibrium shapes of the various BN clusters. The researchers discovered the equilibrium shapes of the BN flakes to be triangular with primarily zigzag edges. The authors also studied hybrid in-plane heterostructures consisting of graphene and hBN. The edge energies of the BN/C interface were determined using the pristine edge values and correcting them via binding energies at the interface. Although expensive, it would be interesting to determine how the estimated values would compare to the values calculated in the traditional way by computing the total slab energy, subtracting the energy of the equivalent bulk material, and assigning the rest to the edges. Nevertheless, the work elucidates how the chemical potential of the species can be used as an additional parameter to control the growth of 2D monolayers (Liu et al., 2011a). In another study, Liu et al. explained the alignment of monolayer hBN on Cu (100) surfaces. The first-principles calculations of total energy versus rotational angles at the initial nucleation stages revealed why hBN orients on the surface in a particular direction, whereas various rotational orientations are observed in the graphene-Cu(100) system. This difference was attributed to the weaker N-Cu and B-Cu interactions than C-Cu interactions. In the future, investigating rotational barriers could inform the stability of rotational orientations observed in CVD-grown structures in greater detail, although it is not necessary to gain insights into the orientational preference, as suggested by Liu et al. Further, the authors observed rearrangements of Cu atoms in the lattice, caused by bonding interactions between the B/N atoms and the substrate, thus pointing to the role of non-van der Waal interactions in the orientation of CVD-grown structures (Liu et al., 2014).

The Ding group studied the CVD growth of hBN on Ni(111), Cu(111), and Rh(111) with the help of DFT (Figure 4B(a)) (Zhao et al., 2015). The authors found that hexagonal rings are favored over other ring shapes such as pentagons or heptagons. Further, the zigzag edge was found to be more energetically favorable than the armchair edge in hBN. In addition, the authors predicted that zigzag Klein edges with dangling atoms are more favorable, as shown in Figure 4B(b). Calculations of the interactions of the edges present in BN nanoribbons (Figure 4B(c)) with the underlying metal substrates revealed that the edges bend in the direction of the substrate, resulting in charge transfer between them. For Klein edges, electron sharing occurred between the edge atoms and the substrate, and between the edge atoms and the bulk hBN atoms. This caused edge passivation, and the charge distribution was delocalized, thereby leading to energetically favorable Klein edges (Zhao et al., 2015). Song et al. reported the growth of single-domain crystalline hBN monolayer sheet using Cu-foil enclosures (Song et al., 2015). The study revealed the dependence of the hBN layer orientation on the facet exposed at the Cu surface. The authors concluded that Cu(111) was the best facet, which was validated by their DFT results. The authors adopted a promising experimental strategy to grow large-area hBN monolayers by suppressing the nucleation density during the growth process. An effective way to reduce the number of nucleation sites is by creating an enclosure of Cu by folding the sheets and using the inner surface as a substrate. Indeed, the reduced feeding rate of the precursor inside the Cu enclosure led to the growth of monolayers on the inner surface in contrast to multilayer growth on the outer surface. Song et al.’s DFT study further revealed that hBN interacts weakly with the metal substrate and the orientation of the monolayer is determined by the edge-substrate interactions. The authors used linear interpolation and symmetry considerations to obtain the binding energies of the edge at angles other than those calculated using DFT. This is an interesting approach as it reduced the number of DFT calculations required but the results still qualitatively matched the experimental data. Overall, this comprehensive work provided insights which could be useful in growing large-area monolayer hBN by controlling the precursor feeding rate to suppress the nucleation density (Song et al., 2015).

Yakobson et al. developed a mechanism for hBN growth using crystal growth theory supported by ab initio calculations (Zhang et al., 2016b). The study postulated that the shapes of the crystals depend on the chemical potentials of boron and nitrogen. The authors used a “nanoreactor”-based approach to study growth kinetics by analyzing the edge growth of BN and showed that the equilibrium shapes of the CVD-grown islands differ from the triangular or hexagonal shapes reported experimentally. This study was one of the first atomic-scale investigations of the CVD growth of 2D hBN (Zhang et al., 2016b). Another work modeled the adsorption and energetics of the building species of hBN layers on Ni(111) and Ni(211) (Liu et al., 2017). The reactive force field (ReaxFF) formulation was used to calculate the Ni-B and Ni-N pair interactions. The study revealed an atomistic picture of the potential energy changes involved in the nucleation and growth of hBN. Linear BN chains were found to initiate the nucleation process and lead to branched and hexagonal shapes. The formation of hexagonal shapes was found to increase with an increase in growth temperature as shown in Figure 4C (Liu et al., 2017). In other work, Fu and Zhang calculated the formation energies of various edges in hBN sheets using self-consistent-charge density functional tight binding (SCC-DFTB) theory (Fu and Zhang, 2017). The researchers noted that the zigzag edges terminated by N atoms have the lowest energy and the zigzag edges terminated by B atoms have the maximum energy. This is in agreement with nanopore edges in hBN being terminated with N atoms, rather than B atoms (Govind Rajan et al., 2019). The energy of the armchair edge was found to lie in between. The work showed that truncated-edge rhombic hBN sheets are more probable energetically as previously seen in experiments (Fu and Zhang, 2017). In a recent study, Zhu et al. investigated the nucleation of hBN on Ni(111) using ab initio calculations. The authors considered three possibilities in their simulations during nucleation – chain-like structures, ring structures, and sp^2^-bonded hexagonal structures (Zhu et al., 2021). Four adsorption sites, viz, top, bridge, hcp, and fcc were investigated out of which the hcp and top site was found to be the favored ones. This study provides detailed insights into the nucleation process: BN pair attachment at the N site was found to be more favored than attachment at the B site. Another interesting insight in this work includes the observed instability of other ring structures like tetrahedrons and pentagons, which contrasts with the edge reconstructions observed in free standing monolayers. The symmetry of the hBN clusters was found to be the most important factor in determining the stability of the structures. In total, this work provides an excellent methodical treatment of the nucleation of 2D monolayers and substrate effects (Zhu et al., 2021).

To conclude, our summary of work in this field (Table 2) indicates that there is a lack of theoretical understanding regarding the chemistry of growth of hBN layers, and how it influences hBN nucleation and growth during CVD on various substrates. Moreover, a comparison of Tables 1 and 2 indicates that there is a lack of studies focused on growth and transport modeling with the emphasis almost solely on ab initio investigations. In this regard, future KMC, reactive MD simulations, and phase-field modeling could offer valuable insights into the chemical growth mechanism of hBN. For instance, similar to the case of graphene, one needs to investigate whether BH_*x*_ and NH_*x*_ clusters, or even mixed B_*x*_N_*y*_H_*z*_ clusters, can participate in the growth mechanism.

**Table 2:** Summary of the studies addressing different focus areas in hBN CVD growth using various theoretical methods

Focus Area ↓	Method →	
	DFT	MD
Nucleation and Growth	Liu et al. (2011a); Zhao et al. (2015); Song et al. (2015); Z. Zhang et al. (2016b); Song et al. (2016); Zhu et al. (2021)	Liu et al. (2017)
Role of the Substrate	Fu and Zhang (2017); Zhao et al. (2015); Song et al. (2015)	

## Modeling of TMD CVD growth

### Experimental background

TMDs are 2D materials with the general formula *MX*_2_, where *M* is a transition metal (e.g., molybdenum (Mo) or tungsten (W)) and *X* is a chalcogen from group 16 of the periodic table (e.g., sulfur (S), selenium (SE), or tellurium (Te)). TMDs are semiconducting materials, with a bandgap in the visible range, and have found applications in optoelectronic devices (Mak and Shan, 2016; Zeng and Liu, 2018) as well as membrane separations (Heiranian et al., 2015; Hirunpinyopas et al., 2017). As opposed to graphene and hBN, there is substantial structural complexity in a monolayer TMD where the metal (*M*) atoms are sandwiched in between the chalcogen (*X*) atom layers. This presents a unique challenge in terms of understanding the separation of the metal and chalcogen atoms from their respective precursors and their incorporation into their corresponding lattice positions. In this regard, various diffusion processes affect the synthesis in different ways – *M* and *X* adatom diffusion affects the growth rate and nucleation density of TMD layers whereas their edge diffusion influences the continuity and shape of the TMD domain. With respect to the vapor-phase synthesis of TMDs, Najmaei et al. synthesized few-layered MoS_2_ using CVD in the year 2013 by the reaction between vaporized MoO_3_ and sulfur. The reaction temperature was maintained at 850**°**C and insulating SiO_2_/Si were used as substrates. The optimum pressure range was found to be 4–10 kPa, wherein the triangular domains coalesced to form large-area sheets of MoS_2_ (Najmaei et al., 2013). Other precurors used for MoS_2_ synthesis include MoCl_5_, Mo(CO)_6_, and H_2_S. Studies have also focused on the role of vapor-phase composition in modulating the growth of MoS_2_. Particularly, the hydrogen-free CVD growth of MoS_2_ on silicon was examined by Wang et al. using SEM and atomic force microscopy. The study found that the location of the Si substrate influences the shape of the domains from triangular to hexagonal configurations. The authors attributed the change in the shape of the crystals to the spatial variation in the Mo:S ratio on the substrate (Wang et al., 2014a).

Other TMD materials that have been synthesized via CVD include MoSe_2_, WSe_2_, and WS_2_. Wang et al. synthesized molybdenum diselenide (MoSe_2_) under ambient pressure conditions. The smaller band gap and higher mobility of electrons make the selenides a better choice for device applications (Wang et al., 2014b). Another study examined the role of hydrogen in the CVD growth of TMDs using ADF-STEM. The authors observed that in the presence of hydrogen gas MoSe_2_ monolayers were the dominant product. The stable edge types included the ZZ-Mo, ZZ-Se, and ZZ-Se-derived Klein edge with dangling Mo atoms (Wang et al., 2018). Liu et al. studied the growth of WSe_2_, which has important applications in mechanical, electronic, and optical devices (Liu et al., 2015). WO_3_ and Se powder were used as the precursors and the growth was performed on Si/SiO_2_ substrates. A rise in temperature led to the formation of WSe_2_ domains followed by geometric shapes which included incomplete triangles and hexagons with various edge structures (Liu et al., 2015). In other work, Chen et al. observed that 2D WSe_2_ grew in a spiral manner driven by screw-dislocation defects (Chen et al., 2014). CVD growth aided by sulfur resulted in pyramidal structures. The sulfur partially reduces the WO_3_ precursor which played a critical role in the growth process. The mechanism of growth and generation was studied in detail (Chen et al., 2014). Subsequently, Kang et al. synthesized WS_2_ monolayers from WO_3_ using hydrogen gas and sulfur via reduction and subsequent sulfurization. At lower hydrogen pressure, the SiO_2_ substrate was etched due to insufficient reaction with WO_3_ whereas, at higher pressures, the WS_2_ layer was etched (Kang et al., 2015).

### Progress and prospects on the theoretical front

**Figure 5:**
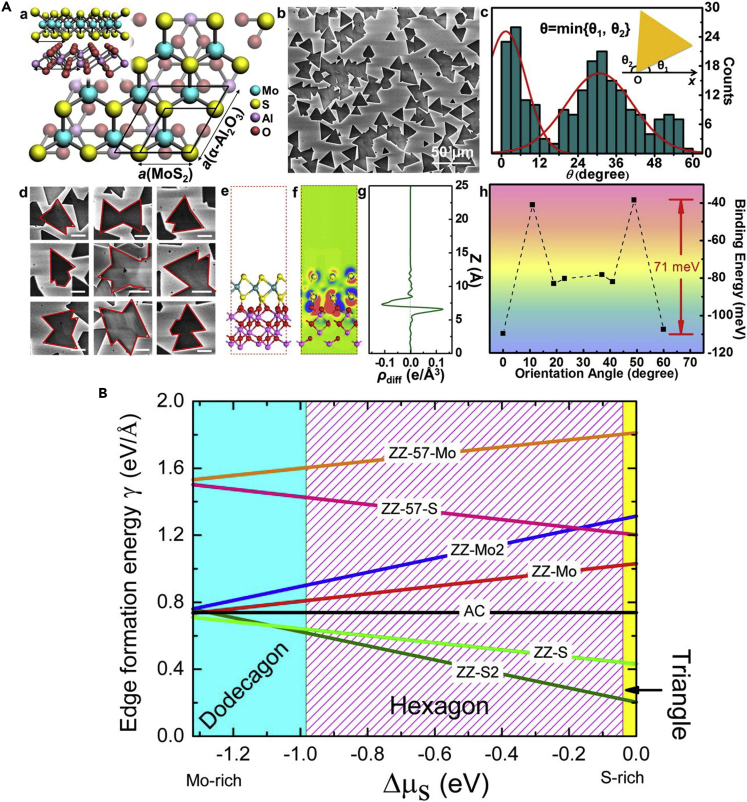
Modeling and experimental studies focused on understanding the CVD synthesis of MoS_2_ (A) Growth of MoS_2_ on sapphire. (a) Side and top views of MoS_2_ on sapphire substrate. (b) SEM imaging of the MoS_2_ flakes formed. (c) Distribution statistics of the orientation angle of the triangular MoS_2_ flakes that are formed. (d) SEM imaging of polycrystalline MoS_2_ flakes. (e) Side view of the optimized structure of MoS_2_ on sapphire. (f) Contour plot of the charge density difference between MoS_2_ and the substrate. (g) Averaged charge density difference in the direction perpendicular to the surface, at zero-degree rotation. (h) Plot of the binding energy versus relative orientation between a MoS_2_ flake and the substrate using DFT calculations; the dips in energy match with the peaks in panel (c).Adapted with permission from Ji et al. (2014). Copyright © 2015, American Chemical Society. (B) Plot representing the stable shape of MoS_2_ domains based on the Wulff construction method. The formation energies of zigzag, armchair, and other edges are plotted versus the chemical potential difference. Adapted with permission from Cao et al. (2015). Copyright © 2015, American Chemical Society

In a combined experimental-modeling study, Van der Zande et al. studied the lattice orientation, morphology of the edges, and crystallinity of the triangular islands formed during the CVD growth of MoS_2_. The authors found that mirror twin boundaries are formed along with form-faceted tilt boundaries, when the island crystals merge together via eight and four membered rings (van der Zande et al., 2013). In this study, solely DFT calculations were used to obtain the relaxed edge geometries, leaving scope for future studies to consider grain boundary formation via KMC and reactive MD simulations. Ji et al. investigated the CVD growth of MoS_2_ on sapphire (0001) substrates (Figure 5A(a,e)) (Ji et al., 2014). The formed domains (Figure 5A(b,d)) were aligned in two directions (Figure 5A(c)) in agreement with their DFT calculations. The binding energies at different angles of rotation (Figure 5A(h)) indicated that there is a minor contribution from vdW interactions, and that the electrostatic interactions play a dominant role in the binding interactions (Figure 5A(f,g)). The controlled growth of MoS_2_ provided a remedy to the problem of misorientation-related polycrystalline CVD growth (Ji et al., 2014). Furthermore, the success of DFT calculations in rationalizing experimental observations of MoS_2_ CVD synthesis promises to provide further mechanistic insights into the growth of 2D materials, in the future.

**Figure 6:**
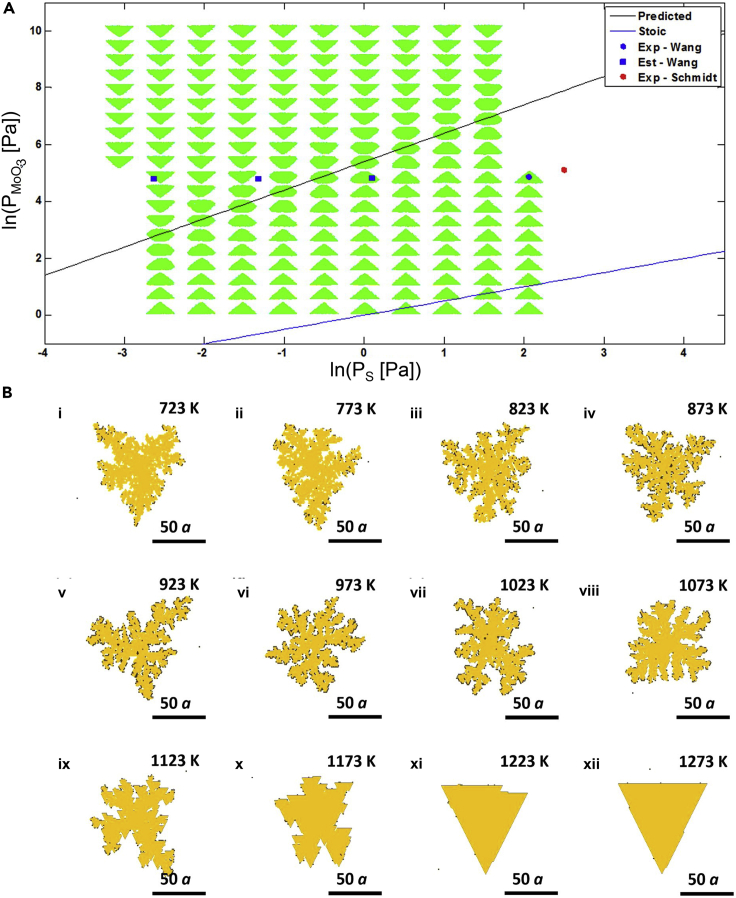
Results from KMC simulations of TMD growth (A) Log-log plot of the partial pressures of MoO_3_ and sulfur, representing a “kinetic phase diagram” for MoS_2_. The crystal shapes obtained in KMC simulations at 700°C are indicated on the plot. Adapted with permission from Govind Rajan et al. (2016). Copyright © 2016, American Chemical Society. (B) Depiction of the morphology of WSe_2_ domains with changing temperature at a fixed flux and chalcogen to metal ratio. (i) through (xii) indicate the various temperatures considered, depicting a transition from dendritic structures to regular triangular structures. Adapted from Nie et al. (2016). Copyright © 2016, IOP Publishing, Ltd.

Cao et al. inferred using DFT calculations that the chemical potentials of Mo and S atoms play a critical role in determining the edge structure and shapes of MoS_2_ crystals (Cao et al., 2015). The authors calculated the edge formation energies as a function of the chemical potential difference to determine the most-stable edge morphology (Figure 5B). They subsequently used the Wulff construction method to reveal the evolution of the various shapes of MoS_2_ domains – dodecagonal to hexagonal and triangular based on the chemical potential difference, as shown in Figure 5B (Cao et al., 2015). Later, Govind Rajan et al. formulated a theory for describing TMD growth for the first time (Govind Rajan et al., 2016). A thermodynamic and kinetic model was constructed from the reactor and reaction parameters that are relevant to growth, shape, size, and edge structure. The development of a transport model allowed the theory to predict growth outcomes observed in multiple experimental studies. The authors observed exceptional agreement between the model and experimental results, with respect to the shape and size evolution of MoS_2_ flakes across the reactor length. Finally, the model was translated into a "kinetic phase diagram" (Figure 6A) using a simplified theory which could be used to achieve controlled TMD growth (Govind Rajan et al., 2016). The kinetic phase diagram allows the prediction of the MoS_2_ flake shape obtained in a CVD reactor, as a function of the precursor partial pressures.

Subsequently, Nie et al. studied the growth of WSe_2_ on graphene using KMC simulations and ab initio calculations (Nie et al., 2016). The KMC simulations involved detailed description of each of the plausible events during growth, such as, precursor diffusion, adsorption, desorption, formation of bonds, and edge diffusion of atoms. The formation of WSe_2_ monolayers was considered as a function of the growth temperature, inlet flux, and chalcogen to metal ratio. The layer stoichiometry and defect density were used to characterize the WSe_2_ domain quality. The domains formed in the simulations were either compact or branched (fractal-like). Specifically, at lower chalcogen conditions, a compact triangular morphology with rough edges and having a higher density of defects was reported. The morphology evolved from dendritic (between 723 and 923 K as shown in Figure 6B(i-v)) to fractal (between 973 and 1123 K as shown in Figure 6B(vi-ix)) and eventually to compact (between 1173 and 1273 K as shown in Figure 6B(x-xii)). The study also developed a domain morphology-based phase diagram which could be used to establish new strategies for developing TMD domains of larger size and better quality (Nie et al., 2016). In another work, Nie et al. carried out KMC simulations of the growth of van der Waals heterostructures of TMDs (Nie et al., 2017). The authors considered the effect of various parameters, such as the stacking sequence, the rates of vacancy and edge diffusion, as well as the ratio of the metal and chalcogen precursors. The authors only considered homogeneous nucleation of the TMD in their model, while investigating the competitive effect between lateral and vertical growth, but heterogeneous nucleation and nucleation assisted by defects can influence the growth process. Thus, there is scope for further investigation to incorporate the effects of other types of nucleation. Nonetheless, the study provided critical insights into the complex growth process of TMD heterostructures and suggested strategies to improve the precision of the growth process (Nie et al., 2017). Later, Caturello et al. investigated the formation of nanoflakes of MoS_2_, MoSe_2_, and MoTe_2_ via DFT calculations (Caturello et al., 2018). The authors observed that nanoflakes of certain sizes were more stable, thereby providing insight into how the nucleation of these materials may occur during their synthesis. The authors tested a “tree-growth protocol” incorporating a modified Euclidean-distance similarity metric while generating new stoichiometric MoX_2_ nanoflake configurations for trial and testing. It is interesting to note that the global minimum configurations reveal a mixture of tetrahedral and square pyramidal geometry for a small number of Mo atoms leading to an elongated 1D structure. Beyond 6 Mo atoms, the formation of 2D nanoflakes was shown to occur for various chalcogen atoms. However, while screening the initial nanoflake configurations for a global energy minimum, the authors set the cutoff similarity parameter at a value which considered 10n configurations, where n is the number of Mo atoms in the nanoflake. This is a heuristic screening criterion put in place and it could be useful to investigate if changes in this criterion can lead to the discovery of more stable nanoflake configurations (Caturello et al., 2018).

In a study focused on the chemistry of TMD growth, Xuan et al. investigated the CVD of WSe_2_ using W(CO)_6_ and H_2_Se as precursors (Xuan et al., 2019). The study modeled the physicochemical steps of the vapor-phase synthesis in a horizontal chamber with cold walls. The critical reactions were determined using quantum-mechanical calculations, thereby providing a training set for parametrizing a ReaxFF model. The important reaction pathways along with their energetics were determined using the developed force field. Further, a CFD simulation of the chamber was performed using various kinetic, thermal, and transport parameters. The developed model thus afforded better understanding of the experimental conditions required and the growth regime achieved for WSe_2_ (Xuan et al., 2019). In another such study, Xue et al. used ab initio calculations to examine the growth of TMDs. The study postulated that one-dimensional Mo_*x*_S_*y*_ chains grow initially on the Au (111) surface (Xue et al., 2018). The stoichiometry of the clusters played an important role during the growth of clusters as suggested in earlier studies. The authors concluded that once the number of Mo atoms exceeded 12, a stable 2D layer could be formed. This number is slightly higher than what has been previously reported in literature. The authors addressed two very important processes: the coalescence of smaller clusters into larger ones and the transformation of a one-atom thick structure to a three-atom thick one. At low coverages of the substrate surface, MoS_3_ clusters were found to be more dominant. In Mo-rich conditions, MoS_2_ clusters became more prevalent than the MoS_3_ clusters. This work provided important insights into intralayer and interlayer interactions and the competition between them (Xue et al., 2018). Nevertheless, investigating the nucleation process in further detail can help shed light on the initial stages of the CVD process. Chen et al. were the first to study the ultrafast growth of TMDs while investigating the CVD growth of WSe_2_ (Chen et al., 2019). Two possible pathways were proposed – quick edge attachment followed by ultrafast diffusion along edges and quick kink nucleation followed by ultrafast propagation of kinks. Their KMC and ab initio calculations revealed that the second path reproduced experimental features and energetics consistently and that the first path is not viable due to a greater barrier for edge diffusion. The authors found that Se dimers attached to the W-terminated ZZ edge and W atoms attached to the Se atoms at the edge. These attachments generated armchair sites of Se atoms thus forming a kink. The W atoms then bonded with the Se atoms at the kink generating more energetically favorable Se sites, which were ultimately occupied very quickly due to the absence of an energy barrier. This investigation thus revealed important insights on the limiting nature of the kinks in CVD growth of the TMDs. The authors however investigated the pathways starting from an initial circular nucleus of the TMD domain. It would be useful to study the same process considering various nucleate shapes, thus providing further scope for research (Chen et al., 2019). Narayanan et al. optimized the various parameters for controlling the CVD growth of TMDs, such as the temperature, carrier gas flow rate, and the position of the substrate, using MoS_2_ as a model system (Narayanan et al., 2019). The experimental setup consisted of MoO_3_ powder in an alumina boat and sulfur powder in a boat present upstream from the MoO_3_ boat at a fixed distance. The authors found out that a promoter, i.e., a coating of a drop of NaCl solution on the substrate, at an optimal concentration enhances CVD growth of 2D materials. This aspect may be examined in the future from a computational perspective, by investigating if aqueous salt solutions can increase the nucleation rate of 2D materials. The authors suggested a sequence of steps to optimize CVD growth processes – first, an ample quantity of precursors should be used to determine the appropriate temperature; subsequently, the position of the substrates/boats should be optimized for different flow rates, at the optimal temperature (Narayanan et al., 2019). The effect of such parameters should also be examined in modeling and simulation efforts.

To conclude this section, the various theoretical studies on TMD CVD synthesis are summarized in Table 3 according to the different issues addressed in each study. We note a lack of studies using reactive MD and CFD modeling in this area, a gap which could be addressed in the future. In terms of focus areas deserving more attention, studies of the nucleation process and the role of the substrate also need to be undertaken.

**Table 3:** Summary of the studies addressing different focus areas in TMD CVD growth using various theoretical methods

Focus Area ↓	Method →			
	DFT	MD	KMC	CFD/Phase-Field Modeling
Nucleation	Caturello et al. (2018); Xue et al. (2018); Chen et al. (2019)		Chen et al. (2019)	
Growth	van der Zande et al. (2013); Ji et al. (2014); Govind Rajan et al. (2016); Cao et al. (2015); Nie et al. (2016); Nie et al. (2017); Caturello et al. (2018); Xuan et al. (2019); Chen et al. (2019)	Xuan et al. (2019)	Govind Rajan et al. (2016); Nie et al. (2016); Nie et al. (2017); Chen et al. (2019)	Xuan et al. (2019)
Role of the Substrate	van der Zande et al. (2013); Ji et al. (2014); Xue et al. (2018); Nie et al. (2017)			
Transport Modeling	Govind Rajan et al. (2016); Xuan et al. (2019)		Govind Rajan et al. (2016); Xuan et al. (2019)	Xuan et al. (2019)

## Modeling the CVD growth of other 2D materials

Although techniques for the vapor-phase synthesis of newer 2D materials, such as silicene, germanene, MXenes, phosphorene, and borophene are still under development, there have been some theoretical studies exploring the CVD growth of these materials. An early study by Takeda and Shiraishi revealed the theoretical possibility of 2D material layers made up of Si and Ge (Takeda and Shiraishi, 1994). With the success of conventional 2D materials like graphene, hBN, and TMDs, researchers started investigating other elements (e.g., silicon, germanium, and tin in group 14 and phosphorus, arsenic, and antimony in group 15) and their prospects in 2D materials chemistry. Shu et al. studied the growth of 2D silicene on Ag(111) using DFT and nucleation theory (Shu et al., 2013). Ground state-clusters of SiN were found to undergo a structural change from non-hexagonal to hexagonal shapes when the cluster size became 22. Parameters such as diffusion barriers, size of the nucleus, and the nucleation barrier and rate were investigated to understand the mechanism of growth. The authors concluded that silicene could be synthesized at a comparatively low temperature (around 500 K). The nucleation rate was studied as a function of both the difference in chemical potential (Δμ) and temperature (T). Higher temperatures above 500 K and larger Δμ (>0.1 eV) resulted in higher nucleation rates leading to creation of numerous silecene nuclei, which in turn introduced larger number of defects in the grown sheet. A lower nucleation rate may therefore be used to obtain silicene with lesser number of defects. Hence, a lower growth temperature was proposed (Shu et al., 2013). In another study, Meng et al. observed using ab initio calculations that silicene has a buckled conformation on Ir(111), which explained the formation of buckled geometries found in experiments (Meng et al., 2013). The buckled geometry indicates the presence of interactions between the silicon layer and the substrate. In this regard, electron localization function (ELF) calculations reveal regions of charge localization in the system and thus help in gaining insights on inter-material interactions. The ELF results of Meng et al. indicated chemical interactions between every pair of silicon atoms and electron localization at top-bridge and fcc-bridge pairs revealed covalent bonding between the Si atoms. The ELF value was much smaller between the Si-Ir atom pairs indicating the absence of electron pairing. We therefore can gain deep insights into the bonding characteristics of the 2D monolayer and its interactions with the substrate via ELF calculations and researchers can use ELF to effectively gauge the inter-atomic interactions during CVD growth on various substrates (Meng et al., 2013). Cherukara et al. studied silicene growth on Ir(111) with the help of MD simulations (Cherukara et al., 2017). The first step involved cluster formation via surface diffusion, subsequently leading to larger rings with the addition of Si atoms. Finally, a continuous sheet was formed due to coalescence of clusters, although several defects were observed. The authors inferred a correlation between the formation of atomic islands and the substrate temperature. The mobility of atoms increased on increasing the temperature and therefore the clusters had a larger probability to come into contact and merge with one another. Bridge formation via dangling bonds or attachment of smaller clusters or adatoms also led to coalescence of the islands. The presence of point and extended defects in the grown monolayer was attributed to the low energy of formation of the defects in silicene (Cherukara et al., 2017). Future studies can investigate the formation of defects during the CVD synthesis of various 2D materials, an aspect that we also highlight later in the paper.

With respect to MXenes, Sang et al. studied the layer-by-layer growth of TiC. Ti_3_C_2_ MXene was used as both the substrate and source, thus resulting in homoepitaxial growth (Sang et al., 2018). The authors observed that TiC layers formed above 500°C on the defunctionalized surface of the MXene to produce Ti_4_C_3_ and Ti_5_C_4_. First-principles calculations augmented by force-bias Monte Carlo and MD simulations were employed to elucidate the growth mechanism and energetics of the process. This work provides a firm theoretical basis for the development of synthesis methods for monolayer transition metal carbides (Sang et al., 2018). In another work, Zhu and David studied the electronic properties, stability, and equilibrium structure of blue phosphorous using DFT calculations (Zhu and Tománek, 2014). The stability of this material was found to be comparable to that of black phosphorous, which is the most stable form of phosphorous, and the stacking of the layers was determined to be similar to that of graphene. The study concluded that CVD growth would be possible on lattice-matched substrates, as well as by stretching the black phosphorous lattice (Zhu and Tománek, 2014). Zhao et al. investigated the growth of blue phosphorous on various noble metal substrates. The authors studied the possibility of forming extended monolayers, rather than a 2D alloy network with metal atoms that is commonly observed in epitaxial growth of blue phosphorous on Au (Zhao and Li, 2021). The (111) facet of four substrates – Au, Ag, Pt, and Pd – were tested for monolayer growth. With the help of DFT and charge density difference calculations, the authors showed that Ag(111) is the only appropriate substrate to grow a blue phosphorous monolayer which can be attributed to the greater strength of P-P bonds than P-Ag bonds, resulting in large clusters. The transition from 1D structures to 2D monolayer islands occured at a cluster size of 19. AIMD simulations of the growth of phosphorene on Ag(111) proved that the configuration of the atoms is preserved at relatively higher temperatures, indicating the stability of the growth process. The work thus presented a systematic methodology to test the growth of blue P on substrates and to carry out a static and dynamic stability study of the structures (Zhao and Li, 2021). Qiu et al. studied the CVD growth of 2D black phosphorene on tin (Sn) catalyst and blue phosphorene on Ag and Au using ab initio calculations (Qiu et al., 2018a). The study found out a large binding energy between the metal substrate and the 2D phosphorene layer which suggested that exfoliation/transfer of the grown layer to other substrates might not be possible for various applications. The authors showed via DFT calculations that black phosphorene, blue phosphorene, and γ-phosphorene can be grown using CVD methods (Qiu et al., 2018a). However, the study did not discuss the chemistry of the reactions that could be used to synthesize phosphorene. In this regard, the phosphorus-containing precursors used to grow phosphorene are presumed to be toxic and difficult to handle (Khandelwal et al., 2017). Hence, there is a need for modeling the CVD growth chemistry *a priori* so that safe and robust synthetic routes can be predicted for experimentalists to synthesize phosphorene.

Finally, with respect to the synthesis of germanene and borophene, there are no modeling studies exploring their growth, in the literature. On the experimental front, Mazaheri et al. were the first to grow single layer borophene by CVD from diborane (B_2_H_6_) pyrolysis (Mazaheri et al., 2021). (Previous studies mainly used molecular-beam epitaxy as the method of choice for the synthesis of borophene.) The rate of deposition and the effect of temperature and pressure on the CVD growth were studied and a comprehensive analysis of the phase and morphology was presented. SEM imaging was done to characterize the morphology of the grown borophene sheets. TEM was used to study the lattice structure (hollow hexagons) of the grown islands. The authors found a temperature of 830–860 K to be most optimal for growth on aluminum substrates (Mazaheri et al., 2021). d’Acapito et al. studied the growth of germanene on epitaxial AlN supported by Ag(111) experimentally, although no simulation studies have explored the CVD synthesis of this material (d’Acapito et al., 2016). From the above discussion, it is evident that not much work has been done on the CVD growth of newer 2D materials and this leaves plenty of room for further modeling studies which can provide important insights to experimental research groups working toward the vapor-phase synthesis of silicene, germanene, MXenes, phosphorene, and borophene.

## Modeling the growth of 2D material heterostructures

The advances made in the synthesis of nanomaterials led to the development of layer-by-layer assembly of 2D materials, allowing *vertical* heterostructures consisting of various 2D materials. Vertical heterostructures are built as a stack of 2D layers held together by weak van der Waals interactions, thereby allowing one to combine the desirable properties of different materials (Geim and Grigorieva, 2013). Apart from out-of-plane heterostructures, *lateral*, i.e., in-plane, heterostructures display unique electronic and photonic properties and allow the formation of junctions of 2D materials. Such heterostructures have paved the way for tuning the functional properties of 2D materials. Nevertheless, there is a lack of understanding regarding how the growth of both in-plane and out-of-plane heterostructures occurs. Note that vertical heterostructures are more readily fabricated, as compared to lateral heterostructures, because strict lattice matching between the various layers is not required. This is because only weak dispersion interactions are operative between the layers, in most cases.

Theoretical studies can play an important role in providing strategies to improve the reliability and robustness of synthesis methods for 2D heterostructures. Before discussing the theoretical advances, we present a few examples of experimental studies that investigated the formation of heterostructures. In an experimental study, Ci et al. were one of the first researchers to report the growth of in-plane heterostructures of hBN and graphene, wherein the domains of hBN and graphene were distributed randomly. The authors observed that the bandgap and the features were contrasting to that of hBN and graphene, indicating that heterostructures can be used to tune the properties of 2D materials (Ci et al., 2010). With respect to out-of-plane heterostructures, Liu et al. studied the growth of hBN on a graphitic surface and on exfoliated graphene using a two-step mechanism. The first step involved graphene growth on Cu foil from liquid carbon. This was followed by the growth of hBN using ammonia borane (Liu et al., 2011b). Ling et al. devised a general growth strategy of in-plane parallel heterostructures from 2D materials and TMDs despite the possibility of lattice mismatch. This methodology could allow researchers to develop functional units for electronic and optoelectronic applications (Ling et al., 2016).

With respect to theoretical work, Fu et al. used DFT calculations to model the growth of MoS_2_/hBN heterostructures on Ni-containing substrates (Ni and Ni-Ga alloy) present on a Mo foil (Fu et al., 2016). First, the researchers ascertained the energetics of decomposition of the precursor for hBN growth, BH_2_NH_2_, concluding that the process is easier on Ni-Ga alloy than on pure Ni. The Mo foil below the alloy surface acted as a source for Mo which underwent sulfurization by H_2_S. The MoS_2_ then followed a “layer-by-layer” growth mechanism on the hBN layer, thereby resulting in a vertical heterostructure. The authors indicated that Ni-Ga alloy has sulfide-resistant properties, thereby preventing poisoning of the catalyst surface (Fu et al., 2016). In another work, Zhang et al. developed a controllable approach to epitaxially grow MoS_2_/WS_2_ heterostructures (Zhang et al., 2021). The structures were synthesized on SiO_2_/Si substrates in confined CVD chambers at atmospheric pressure using MoO_3_ and sulfur as precursors. The experimental growth was complimented with dispersion-corrected DFT calculations of high-symmetry stacking patterns. It was found that the AA and AB stackings were energetically favorable (Zhang et al., 2021). The determination of optimal stacking sequences is important from the perspective of understanding the synthesis of vertical heterostructures.

**Figure 7:**
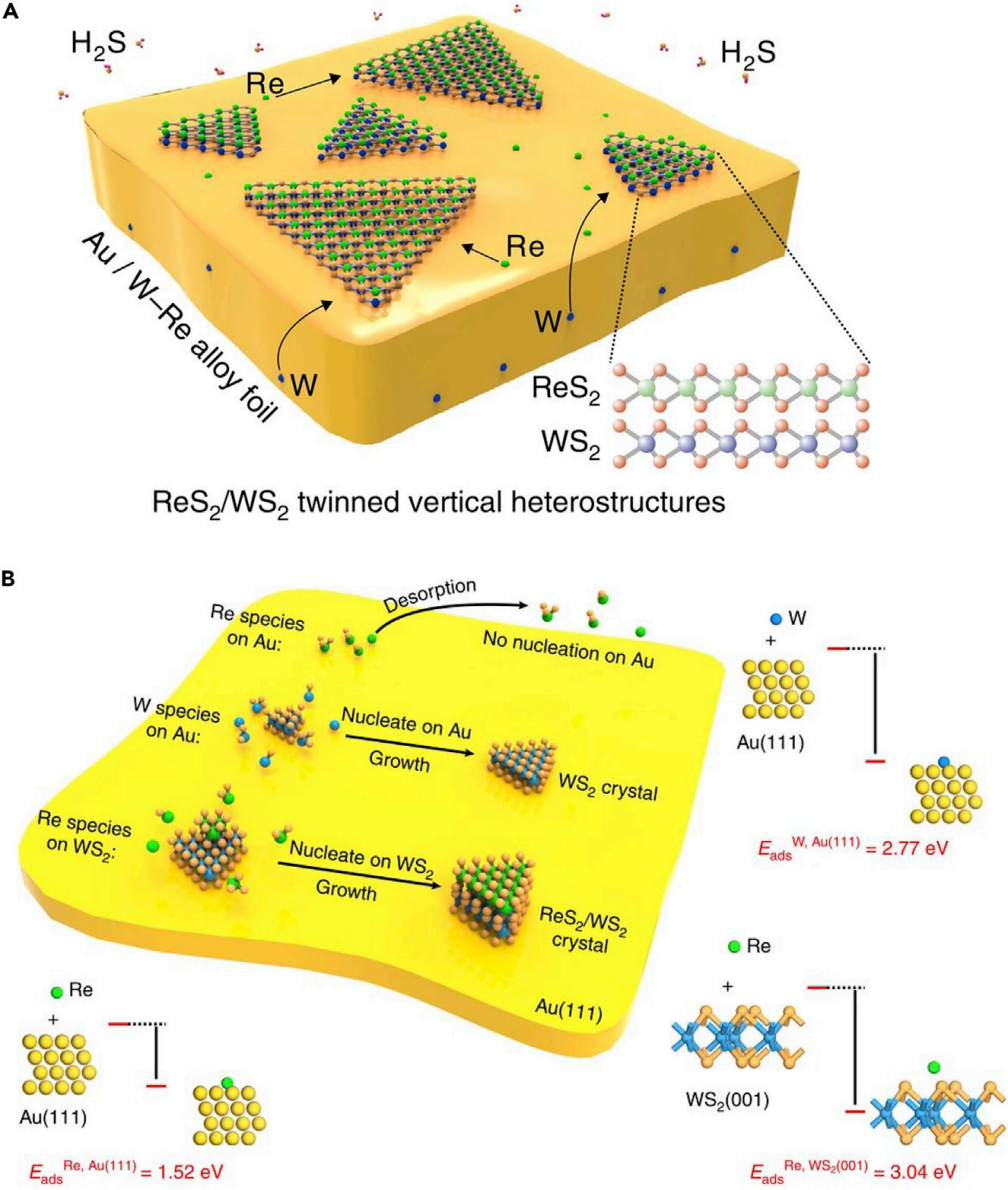
Mechanism and modeling of the growth of an out-of-plane 2D ReS_2_/WS_2_ heterostructure (A) Schematic of twinned growth of ReS_2_/WS_2_ heterostructures on Au substrate with W-Re alloy support (B) Adsorption energies for Re on Au(111), W on Au(111), and Re on WS_2_(001) obtained from theoretical simulations support the twinned growth of WS_2_ and ReS_2_. Adapted from Zhang et al. (2016a). Copyright © 2016, The Authors

The knowledge gap in understanding the mechanism of the growth process of heterostructures presents difficulties in developing scalable synthesis routes for both vertical and lateral heterostructures. In this regard, some theoretical studies have attempted to throw light on the mechanism of heterostructure formation. Zhang et al. utilized a twinned growth strategy to grow vertical heterostructures of ReS_2_/WS_2_ (Zhang et al., 2016a). The authors used DFT to simulate the growth of ReS_2_ and WS_2_ on Au(111) substrate (Figure 7A). The energy for adsorption of W over Au(111) (2.77 eV) and Re over WS_2_ (3.04 eV) were found to be similar, whereas the adsorption energy of Re over Au(111) (1.52 eV) was much weaker Figure 7B). At a growth temperature of 900°C, Re was found to desorb from the Au (111) surface making the nucleation process an unlikely event. WS_2_ nucleated on the surface owing to its higher adsorption energy. Further growth of ReS_2_ on the WS_2_ surface was induced by the high adsorption energy of Re atoms on the WS_2_ domains. Hence, the authors postulated a twinned growth behavior which was validated using various substrates, including foils made from Re, W, and Re-W alloy (Zhang et al., 2016a).

**Figure 8:**
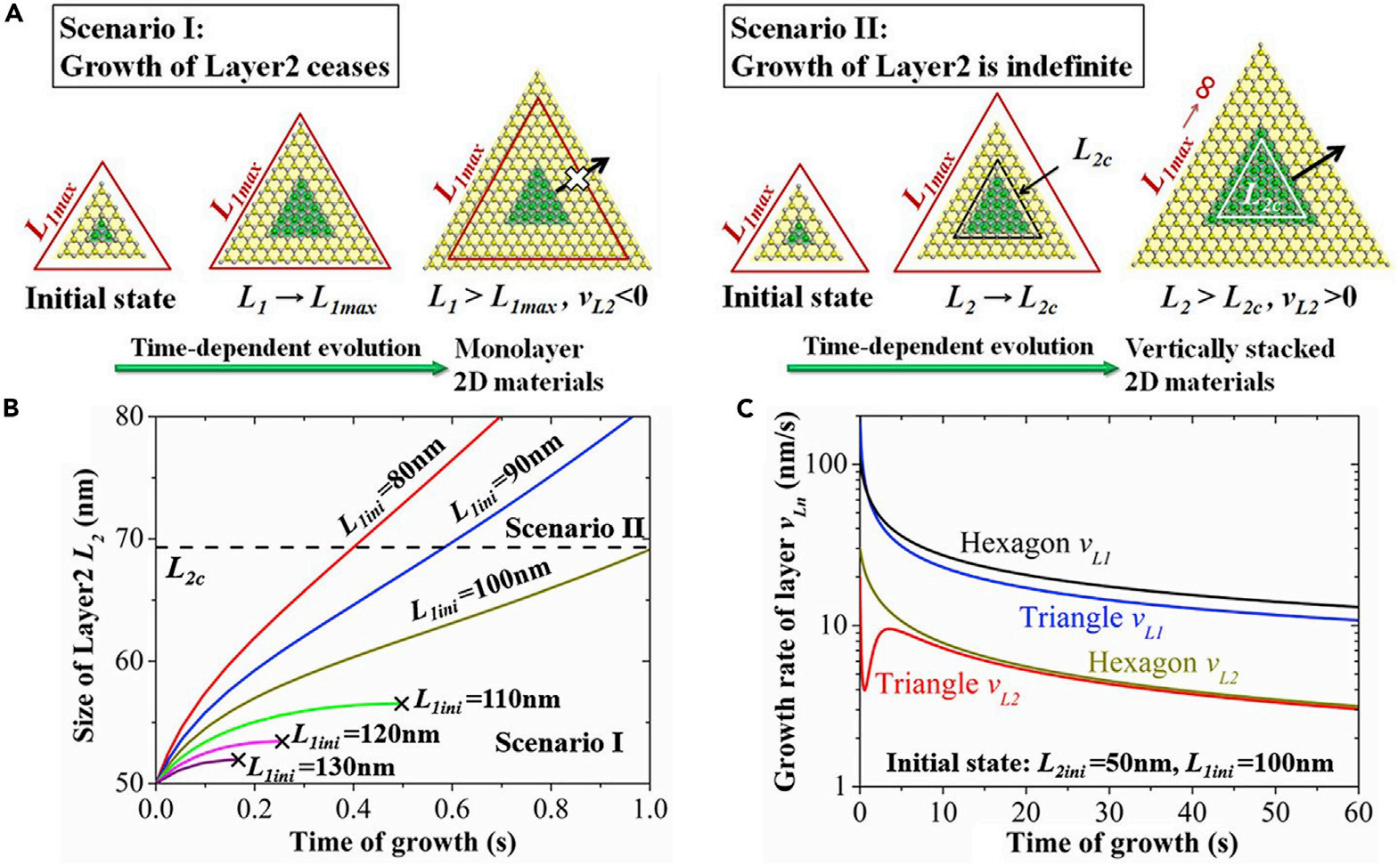
Understanding layer-by-layer versus layer-plus-islands growth (A) Schematic of the adlayer growth with time, depicting island growth of the adlayer (Scenario I) and full growth of the adlayer (Scenario II) (B) Growth of second layer versus time at different initial sizes of first layer. (C) Hexagonal and triangular growth of individual vertical layers with time. Adapted with permission from Ye et al. (2017). Copyright © 2017, American Chemical Society

Ye et al. investigated the thermodynamics and kinetics of heterostructure growth. The vertical growth was analyzed using the finite element method (FEM) to solve the diffusion equations and the energetics of growth were calculated using DFT (Ye et al., 2017). The authors postulated two possibilities for time-dependent vertical growth: (i) the first layer stopping the growth of the second layer, leading to the formation of a monolayer and (ii) no hindrance to the growth of second layer leading to a vertical structure (Figure 8A). The initial state is instrumental in dictating the growth mode and rate, leading to either island growth (Scenario I) or indefinite growth (Scenario II) of the second layer (Figure 8B). The growth rates of various shapes of heterostructures, including hexagons and triangles, were calculated as shown in Figure 8C. A general growth process was proposed using relevant physical parameters, such as temperature, initial layer size, and adatom flux, which likely play an important role in determining the final structure of the heterostructure (Ye et al., 2017).

Note that lateral heterostructures are primarily synthesized using CVD techniques due to the requirement of atomic-level precision at the edge junctions (Cai et al., 2018); they cannot be readily created using liquid-phase or scotch-tape exfoliation. Bogaert et al. showed that surface diffusion significantly influences the CVD growth of lateral TMD heterostructures (Bogaert et al., 2016). A two-step growth procedure was employed, wherein monolayers and stacked layers of WS_2_ were grown at a temperature of 1100°C. Thereafter, WO_3_, the precursor for WS_2_ growth, was swapped with MoO_3_ at a lower temperature (650–710°C) to grow lateral heterostructures of WS_2_ and MoS_2_. The Mo atoms absorbed at the edges of the WS_2_ island and diffused throughout the island. At lower temperatures (around 650°C), distinctive cores of MoS_2_ were observed, as opposed to a uniform alloy, which was reported at higher temperatures (680–710°C) (Bogaert et al., 2016). Subsequently, Zhou et al. formulated a one-step growth strategy to grow WS_2_-MoS_2_ lateral heterostructures. The sizes of the MoS_2_ cores and WS_2_ shells were controlled by adjusting the ratio of the masses of the precursors used. The size of the heterostructure was modified by changing the temperature of growth (Zhou et al., 2018). In another work, researchers studied the growth of lateral and vertical heterostructures of graphene/hBN on Cu(111) (Makino et al., 2020). The bonds between the graphene and Cu substrate were broken when hBN precursors reached the graphene edges. Subsequently, hBN was formed at the graphene edges with the formation of carbon-boron bonds, leading to hBN-graphene lateral heterostructures. When hBN grew first on metal substrates, triangular islands with zigzag edges terminated by N were formed, with no bonds between the hBN edge and the substrate. Graphene then grew between the hBN layer and the Cu substrate resulting in vertical heterostructures (Makino et al., 2020). Despite these experimental advances on the CVD growth of lateral heterostructures, there is a lack of modeling studies aimed at understanding their growth. Nevertheless, the experimental studies clearly point to the role of temperature and precursor ratios in modulating the synthesis of lateral heterostructures. Going forward, it will be instructive to understand the mechanism of growth and to gain theoretical insights regarding the relative importance of various process parameters, to augment experimental efforts in this direction. Indeed, ab initio calculations and KMC simulations can be combined to better understand the growth process of lateral heterostructures, thereby presenting opportunities for future research. In this regard, the energetics of, and barriers for, bond formation between various 2D materials, can be estimated using DFT calculations or reactive force field simulations.

## Use of machine learning to understand and predict CVD growth

Machine learning (ML) has assumed great importance in modern-day research due to the availability of large amounts of experimental and simulation data, as well as large materials databases. These data can be used to train statistical models, thereby allowing one to make predictions regarding the type of growth that will result, given the process conditions used. Indeed, ML has found many applications in CVD growth, especially in prediction and classification of material morphology, and growth tracking. Note that mainly two types of ML exist, supervised learning and unsupervised learning. In the former, existing data is used to train a specific type of model, e.g., linear regression, random forests, gradient-boosted decision trees, support vector machines, etc., to predict a certain observable. In the latter technique, existing data is used to infer the correlations and connections that may exist within the dependent and independent variables, e.g., using k-means clustering. A common example of unsupervised learning is “clustering” where the datapoints are clustered into various classes based on the underlying features of the dataset. Most of the work carried out in this field thus far has focused on supervised learning, leaving room for using unsupervised learning to categorize and interpret the CVD process in the future.

**Figure 9:**
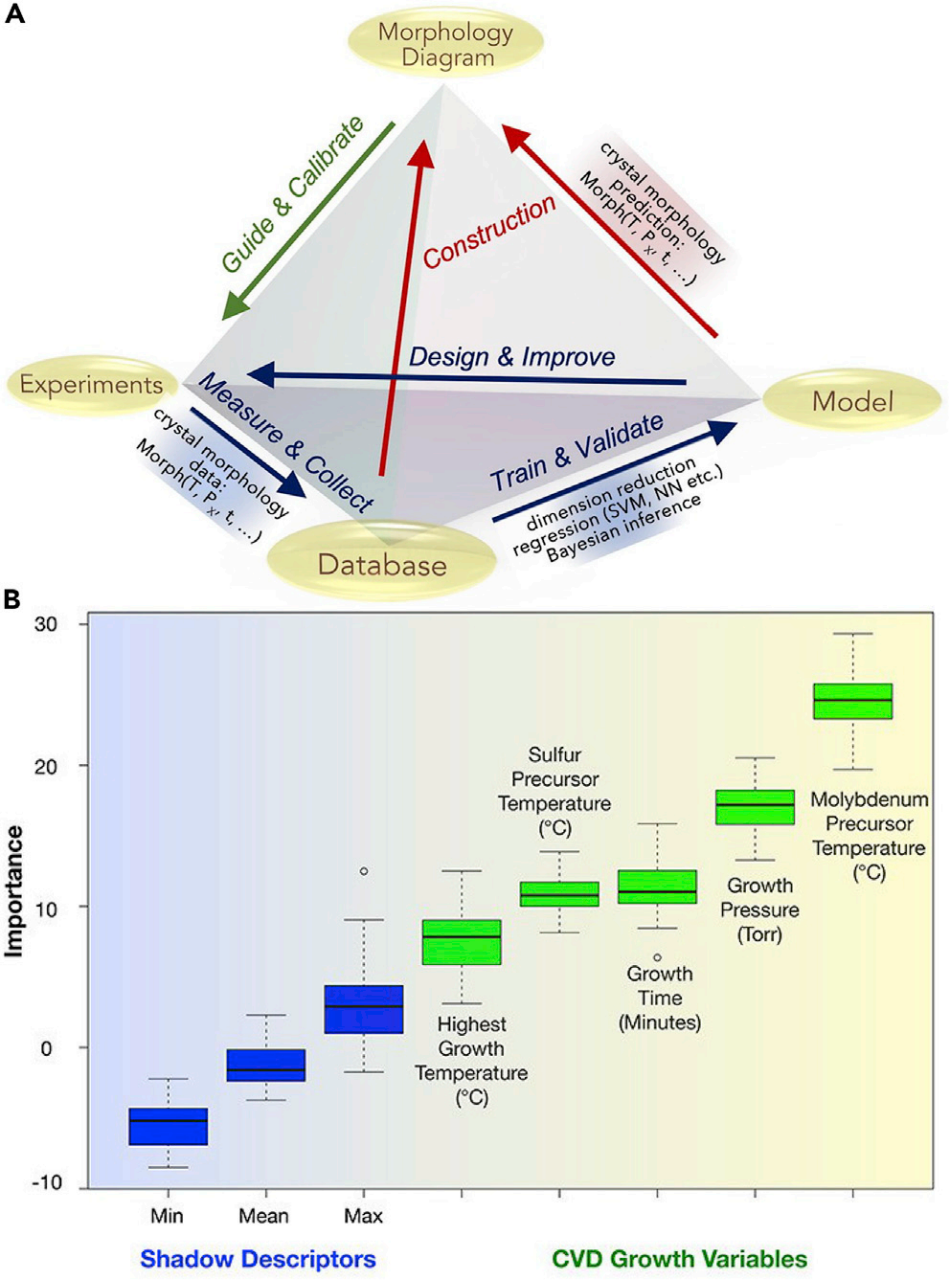
Usefulness of data analysis in understanding CVD synthesis (A) A modeling, experimentation, and database development-based “materials genome” approach to create morphology diagrams of CVD-derived 2D systems. Adapted from Zhang et al. (2020). Copyright © 2020 Elsevier Ltd. All rights reserved (B) Feature importance plot depicting the relative significance of the growth parameters in the order Mo precursor temperature > growth pressure > growth time > sulfur precursor temperature > highest growth temperature. Adapted from Costine et al., 2020. Copyright © 2020, the Authors. Rights managed by AIP Publishing

In terms of previous work, Zhang et al. emphasized the need for a data-based approach, including a combination of experiments and modeling, to overcome the challenge of achieving controllable CVD growth of 2D materials (Zhang et al., 2020). The authors elucidated how morphology diagrams could facilitate validation of hypothesized synthesis mechanisms based on available data. Indeed, large amounts of data on various synthesis conditions and their respective growth outcomes can lead to an unbiased assessment of synthesis routes by invoking the methods of data analytics and ML. A schematic explaining this workflow is shown in Figure 9A (Zhang et al., 2020) and can help in finding underlying relations between growth outcomes and the process parameters (Momeni et al., 2020). As seen in Figure 9A, materials databases and models can be used to create morphology diagrams, and such diagrams can be used to guide experiments. Subsequently, the experimental data could be added to the materials database, leading to further optimization of the ML model.

Ziatdinov et al. trained a deep neural network model using scanning transmission electron and probe microscopy images of graphene samples (Ziatdinov et al., 2017). The atomic coordinates obtained from the deep neural network led to the identification of a variety of defects which were absent from the training set. This approach further led to the interpretation of complex atomic transformations and defects. For example, a single Si dopant defect was observed to transform into a Si dimer. Using the model, the intermediate structures were elucidated which transformed from a 4-fold Si structure to a 3-fold Si structure and vacancy, and finally to a Si dimer structure (Ziatdinov et al., 2017). Krishnamoorthy et al. used a neural network model to analyze the structure and defects of atomic systems (Krishnamoorthy et al., 2020). The researchers used a feature vector containing 436 dimensions from radial and angular symmetry operations for analysis of the local structures. Subsequently, the authors developed a deep neural network model to develop force fields for MD simulations. The use of forward propagation while calculating forces afforded improved prediction speeds as was validated against true values (test set) generated from DFT simulations (Krishnamoorthy et al., 2020). Neural network-based force fields offer possibilities to study the CVD growth of 2D materials involving multiple competing chemical reactions and mechanisms, thereby presenting numerous opportunities for future research. To this end, Wang et al. developed an artificial neural network (ANN) model for first-principles simulations of TMD heterostructures (Wang et al., 2019b). The model was trained using a multitude of configurations and their energies obtained from DFT calculations. The classical MD simulations performed from the trained ANN model elucidated the stability of the heterostructures at a lower computational expense and presents a way for researchers to study other heterostructures which are computationally challenging to model using explicit AIMD simulations (Wang et al., 2019b).

Studies focused on extracting the best growth conditions from ML models have also been carried out. Schiller et al. developed a crowd-sourced database of synthesis routes and experimental findings of CVD growth of graphene (Schiller et al., 2020). Many parameters including catalyst composition, ambient temperature, reactor specifications, and spectroscopy and microscopy results were included in a database and were processed by ML algorithms to predict tangible synthesis routes. This database has the potential to advance the discovery of CVD routes for the synthesis of 2D materials at an accelerated pace (Schiller et al., 2020). Recently, Costine et al. developed an ML-based approach to predict the growth of MoS_2_ using previously unexplored process parameters, using data available from the literature on the CVD synthesis of MoS_2_ (Costine et al., 2020). An unsupervised metric multidimensional scaling (MDS) analysis was performed using the growth variables, namely, Mo and S precursor temperature, maximum temperature of growth, growth time, and pressure. No tangible “favorable regions for growth” were found using the MDS analysis and hence, a supervised ML strategy was applied. The importance of various features was extracted from a random forest ML model (Figure 9B), indicating the Mo precursor temperature to have the most significant effect on the growth process (Costine et al., 2020). Lee et al. tested different processing pathways to reduce uncertainty in the growth of vertically aligned carbon nanotubes (VACNTs) (Lee et al., 2019). Eleven separate growth routes were statistically analyzed from 95 nanotube samples. The authors observed that vacuum baking at 200°C decreased the variability of growth significantly by removing water vapor and volatile components from the reactor. Thereby, a data-driven approach was successfully used by the authors to reproducibly grow VACNTs (Lee et al., 2019).

**Figure 10:**
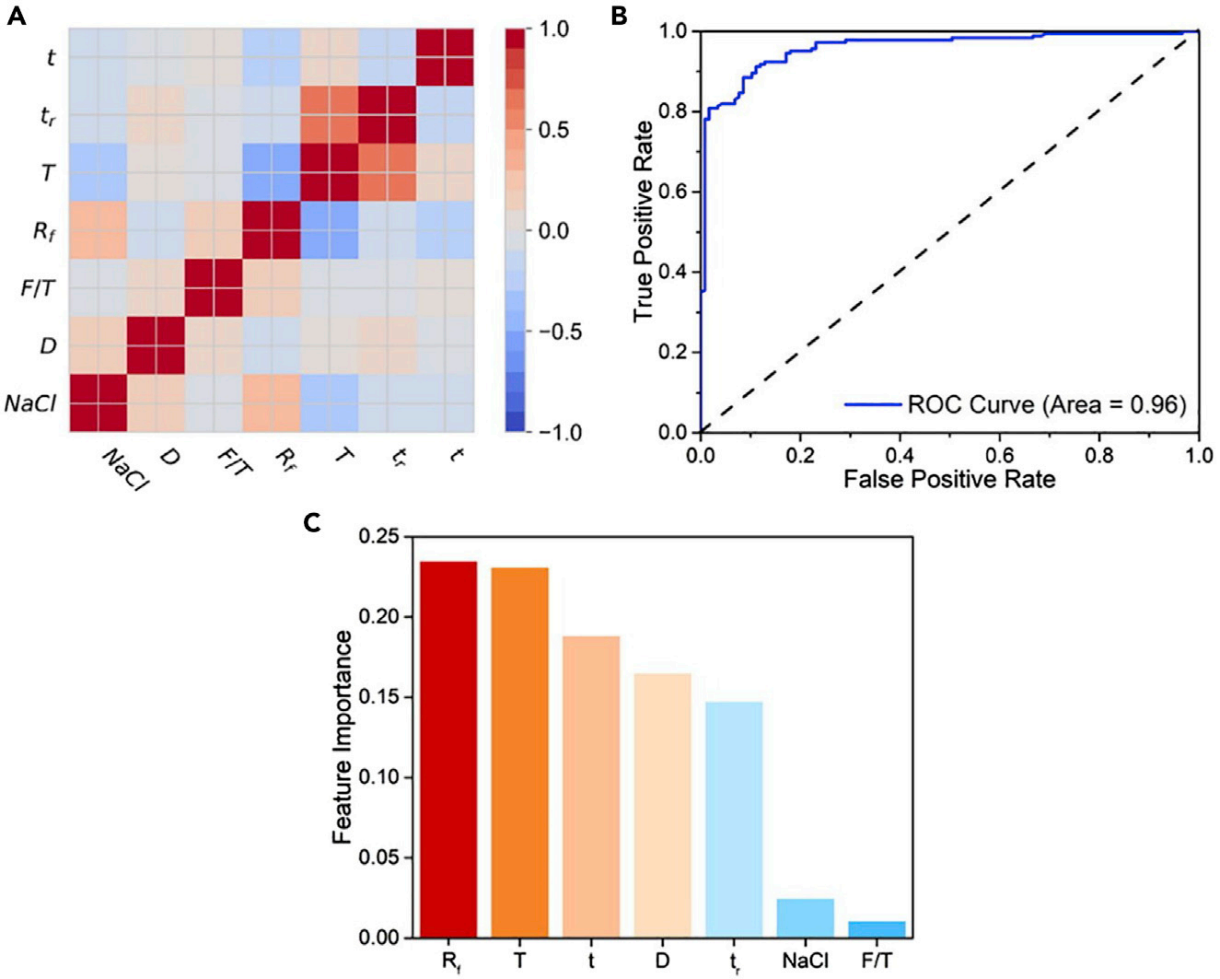
Evaluation of a machine-learning model developed from MoS_2_ CVD growth data (A) Heatmap representation of Pearson’s correlation matrix based on the features selected. (B) Receiver operating characteristics obtained from the XGBoost model. (C) Feature importance plot of the XGBoost model using the consistent SHapley Additive exPlanations (SHAP) method. Adapted from Tang et al. (2020). Copyright © 2020 Elsevier Ltd. All rights reserved

In another study, Xu et al. used a supervised ML algorithm for understanding the CVD growth of high quality WTe_2_ nanoribbons (Xu et al., 2021). The growth parameters were optimized using the trained ML model. The authors concluded from the feature importance study that the flow rate of hydrogen gas and temperature of the reaction were the most critical parameters. Hence, it was observed that augmenting experimental results with ML models can accelerate development of nanoribbons (Xu et al., 2021). In other work, Tang et al. used ML to study the synthesis of MoS_2_ via CVD (Tang et al., 2020). A progressive adaptive model (PAM) was employed, which started with a small dataset initially and was evolved iteratively using feedback loops. The PAM was used to enhance the outcomes of the experimental synthesis procedure. The researchers used a dataset containing results from 300 experiments wherein successful growth was observed 183 times. A binary classification problem was naturally formulated from the available data wherein success was termed the positive class and failure was termed the negative class. Features were divided into two parts – reaction- and process-related. Seven features with a complete dataset were considered for the feature-importance study, which included the gas flow rate, reaction temperature and time, ramp time, distance of the sulfur precursors outside the furnace, and the boat orientation. The mutual information between the features was quantified using Pearson’s coefficients. As seen in Figure 10A, apart from the diagonal entries, the coefficient of correlation between various variable pairs is very low, suggesting that the chosen parameters can be used as unique descriptors, due to the lack of correlation between them. The authors employed a gradient-boosted classifier (XGBoost), support vector machine classifier (SVM-C), naive Bayes classifier (NB-C), and multi-perceptron classifier (MLP-C) to select the best model. The work showed that the XGBoost classifier model performed the best (Figures 10B and 10C) based on training and validation, and thus paved the way for its use to predict results for uncharted process conditions (Tang et al., 2020). Indeed, a large area (0.96) under the receiver operating characteristic (ROC) curve in Figure 10B shows that model can effectively differentiate between two classes, one denoting growth (“can grow”) and the other denoting no growth (“cannot grow”).Further, in Figure 10C, one can see that the flow rate of the gas (R_f_) is the most important parameter. The temperature (T) and time of reaction (t) are the next two parameters in order of importance. The deposition rate is affected by the gas flow rate, the temperature, T determines the reactant vapor pressures, and the thickness of the layers are affected by the reaction time, t.

Liu et al. used reactive MD simulations to study the CVD growth of MoS_2_ from MoO_3_ and S precursors (Liu et al., 2019). Subsequently, the authors used a machine-learning approach involving feedforward neural networks to identify the critical reaction mechanisms. The results from the training of 36,000 simulation datapoints revealed novel growth mechanisms which turned out to be fundamental for augmenting the experimental CVD growth of MoS_2_ (Liu et al., 2019). To summarize this section, ML-driven predictions are less expensive and faster than the traditional ab initio calculations and hence can be effectively used to obtain required information from a vast pool of data quickly. Notwithstanding the growing literature in this area, there is plenty of scope for ML-driven studies geared toward modeling, predicting, and understanding CVD growth to obtain insights for experimentalists to capitalize on.

## Knowledge gaps and future research directions

The growth of 2D materials via CVD is a promising strategy to produce large-area surfaces in a scalable manner. However, there are several knowledge gaps, particularly from a theoretical standpoint, that prevent further advances in understanding and engineering the controlled synthesis of 2D materials. Some of these knowledge gaps are discussed in this section and could provide avenues for future research.

First, the formation of grain boundaries at locations where different 2D material islands coalesce is still not well understood (Yao et al., 2020). In this regard, Wang et al. conducted a combined experimental-theoretical investigation where they examined the coalescence of graphene grains misaligned at an angle of 21.8° using DFT calculations and that of MoS_2_ grains using phase-field modeling, along with*in situ* imaging (Wang et al., 2020). The authors concluded that the 2D material-substrate interaction plays a prominent role in the formation of grain boundaries, with strong adlayer-substrate interactions dictating the orientation of the individual grains. In another study, Li et al. used phase-field modeling to study the formation of grain boundaries in graphene (Li et al., 2018). The authors showed that curved grain boundaries and triple junctions are likely in graphene when nucleation occurs in a random fashion. Chen at al. used KMC simulations to model the kinetics of formation of grain boundaries in MoS_2_ and validated their predictions using high-resolution TEM (HRTEM) imaging (Chen et al., 2019). However, such studies have not been carried out for other 2D materials. Looking forward, several opportunities are available to investigate the merging of grains and the formation of grain boundaries in 2D materials via DFT calculations, KMC simulations, and reactive force field simulations. A related aspect to this work is the computational investigation of the formation of in-plane heterostructures. As was seen in our review of the literature, solely experimental studies have been carried out on this front. Here, DFT calculations can be used to predict the energies of edges formed at the junction of disparate 2D materials and the chemistry of combination of precursors containing different metals and nonmetals, which could then feed into KMC simulations of the growth of in-plane heterostructures.

Second, several experimental studies have recently reported the formation of nanopores in 2D materials during the growth process itself. These nanopores are useful from the perspective of using 2D materials for membrane separation applications. Very recently, Yuan et al. reported the CVD synthesis of nanoporous graphene to produce monolayered graphene membranes demonstrating the highest hydrogen to methane permeation selectivity reported so far (Yuan et al., 2021). The authors observed a larger number of nanopores at a growth temperature of 800°C, as compared to 900°C. From this observation, they inferred that a lower temperature reduces the rate of defect healing to a larger extent than the rate of defect formation, in agreement with DFT results from the literature. Previously, Yuan et al. synthesized nanoporous graphene via CVD, with the level of defects quantified by the D peak in the Raman spectrum of graphene. The authors hypothesized that the number and size of the nanopores formed in graphene depended on the amount of oxygen leaking into the CVD reactor from the atmosphere (Yuan et al., 2018). Other experimental work has also reported the presence of gas-sieving defects in large-area CVD-grown graphene (Huang et al., 2018; Khan et al., 2019), with the latter work employing a benzene-based CVD process. Reduction in the CVD temperature has been successfully used to introduce defects into graphene (Kidambi et al., 2018; Villalobos et al., 2021), by promoting polycrystalline growth. Despite these experimental advances, there is a lack of understanding about how nanopores are formed during the CVD process and how their shape and size may be controlled. Thus, theoretical studies in this direction can be very promising. A KMC-based study by Dutta et al. studied competitive etching and growth of graphene to form nanopores (Dutta et al., 2019). The authors observed the creation of elongated nanopores based on activation barriers for methane dehydrogenation and carbon detachment from the lattice. Such studies may find it beneficial to use the isomer cataloging technique developed by Govind Rajan et al. to distinguish between nanopore shapes in 2D materials using graph theory (Govind Rajan et al., 2019), and thus understand the shapes of nanopores formed during the CVD process. Furthermore, the inclusion of ab initio barriers and reaction rates can make the modeling of nanopore creation during CVD more realistic.

Third, more work is needed to predict the effect of the substrate on 2D material nucleation and growth. Models that can predict the growth mode (i.e., layer-by-layer, island, or layer-plus-island) need to be developed to aid experimental efforts and make them more reproducible. In this regard, imaging techniques coupled with ML have the potential to identify growth modes in an automated fashion to create a library of growth recipes. To this end, recently, Millard et al. characterized CVD-grown WSe_2_ and MoSe_2_-WS_2_ heterostructures via automated photoluminescence imaging and image processing (Severs Millard et al., 2020). Such studies offer great promise and potential for the future. In a theoretical study, Shang et al. combined DFT simulations and calculation of phase diagrams (CALPHAD) modeling to understand the competition between lateral and vertical growth for MoS_2_ on graphene and sapphire substrates (Shang et al., 2016). The authors concluded that the growth of bilayer MoS_2_ becomes favorable as the size of the monolayer increases, with the substrate primarily determining this critical size of the MoS_2_ flake. A related knowledge gap is predicting the conditions that promote monolayer versus multilayer growth, which will have to be addressed to precisely control the number of layers in the grown 2D materials. Very recently, Ahn et al. developed an experimental method to grow layer-controlled MoS_2_ films using MoOCl_4_-based chemistry, with control achieved by simply varying the pressure in the system (Ahn et al., 2021). Future work can explore such ideas from a computational perspective to discover growth strategies to enable layer control for 2D materials. In this regard, Guo et al. formulated a Burton-Cabrera-Frank model based on lattice incorporation energetics, kinetic barriers, vdW interactions, layer edge energy penalties, and adatom entropies, to predict the conditions required for vertical versus monolayer CVD growth of 2D layers of TMDs. The authors used a phase-field/diffuse domain-based method to numerically solve the model. The study performed was parametric, but moving forward, the exact energies could be obtained from ab initio calculations and this can be an avenue for future studies (Guo et al., 2020). In another recent work by Zeng et al., the authors presented the limitation of static DFT calculations in obtaining KMC rates in cases where the substrate melts during CVD. The authors compared the minimum energy path (MEP) model with data from AIMD simulations for graphene growth on the Cu (111) facet. At lower temperatures (300 K), methane dehydrogenation had similar energy barriers as obtained from MEP and AIMD. However, at higher temperatures (1300 K) the structure of Cu (111) drastically changed due to melting, resulting in sampling of different structures in AIMD from those obtained in MEP. The dehydrogenation barrier for larger species (CH_3_ and CH_2_) remained almost same in both the cases but the barrier for smaller species (CH) changed. This behavior was attributed to the steric hindrance encountered in the movement of larger species. The CH atoms were trapped in between the Cu atoms making their local coordination different from that in MEP. On the other hand, the steric hindrance did not allow the larger species to be easily trapped inside Cu cavities and it was difficult for the Cu atoms to reach the C atoms in CH_3_ and CH_2_. Thus, the researchers showed that at higher temperatures (note that CVD is generally a high-temperature process) DFT-based MD simulations are the way to go forward to determine the rates and activation barriers depending on the availability of computational resources (Zeng et al., 2020). Moving forward, accurate estimates of precursor adsorption energies and diffusion rates on the bare substrate and the 2D material, as well as of the growth rates of the 2D layers, via first-principles calculations, are essential to make realistic predictions. Such investigations can also tie into the study of out-of-plane heterostructures of 2D materials, where the competitive nucleation and growth of various 2D layers can be examined.

Fourth, efforts toward modeling the vapor-phase and surface transport inside the CVD reactor will be valuable to understand transport limitations and their effect on the morphology of the grown 2D materials. Indeed, detailed CFD modeling of the reactor could be undertaken to understand the role played by fluid mechanics and mass transfer in the nucleation and growth process. Momeni et al. combined phase-field modeling with simulations of heat and mass transfer inside a CVD reactor to understand the synthesis of MoS_2_ (Momeni et al., 2018). By using accurately determined precursor concentration profiles in their model, the authors demonstrated good agreement between the simulated and experimental morphology of MoS_2_. In another work, Ji et al. quantified the role played by fluid, heat, and mass transport on the CVD growth of hBN on Cu(111), using a CFD simulation coupled with phase-field modeling (Ji et al., 2021). In terms of mass transfer on the surface of the substrate, Li et al. revealed the role played by the Mo concentration in determining whether dendritic or compact growth of MoS_2_ occurs via DFT-supported KMC simulations (Li et al., 2019). By quantifying diffusion and attachment barriers via DFT calculations, the authors accurately modeled surface transport phenomena during the CVD growth of MoS_2_. Despite these studies, more investigations are required to realistically model the nanoscale and macroscale processes occurring simultaneously inside a CVD reactor.

Finally, as discussed in the section on modeling the CVD growth newer 2D materials, computational investigations into the vapor-phase synthesis of silicene, germanene, phosphorene, borophene, and layered oxides (e.g., MoO_3_) will be valuable. In this regard, understanding the chemistry and the reaction mechanism underlying 2D material growth is also essential. CALPHAD-type models can provide potential preliminary clues regarding the possible chemical reactions that can occur during CVD synthesis. These would have to be combined with more detailed DFT calculations of reaction free energies and activation barriers, which could feed into KMC simulations of the growth process. Finally, reactive force field-based MD simulations can also be used for growth mechanism discovery, as was demonstrated for the case of MoS_2_ (Hong et al., 2017). This would, however, involve the painstaking effort of developing reactive force-field parameters using DFT calculations. An alternative approach could be the use of AIMD simulations to probe the synthesis of newer 2D materials.

## Conclusions

In this review, we discussed how modeling and simulation techniques can be used to inform and predict the synthesis of 2D materials via CVD processes. We reviewed how theoretical studies using DFT calculations, KMC simulations, and reactive MD simulations help understand the mechanisms underlying nucleation and growth, as well as optimize reaction conditions, such as temperature, pressure, vapor-phase composition, and carrier gas flow rate, to obtain large-area 2D materials. While DFT calculations provide accurate estimates of potential/free energy surfaces, one often needs to combine them with KMC simulations or use them as input to AIMD or reactive MD simulations to understand the kinetics of vapor-phase nucleation and growth. We discussed several studies focused on modeling the CVD synthesis of various layered materials, such as graphene, hBN, MoS_2_, MoSe_2_, WS_2_, and WSe_2_. Furthermore, we provided a brief overview of theoretical investigations of the vapor-phase synthesis of newer 2D materials, such as phosphorene, silicene, MXenes, and borophene. There is a considerable lack of theoretical mechanistic studies in this area, which may be addressed in the future. We also reviewed the literature considering the synthesis of vertical and lateral heterostructures, identifying the formation of in-plane heterostructures as a topic requiring more research effort. Subsequently, we discussed the use of machine learning strategies to understand, predict, and improve the CVD of layered materials, in conjunction with databases of materials properties and experimental growth outcomes. Finally, we outlined several knowledge gaps that could be addressed by theoretical studies in the future. In particular, the study of grain boundary formation and defect incorporation in 2D materials during CVD synthesis, the prediction of various growth modes and reaction chemistries as a function of reactor conditions, modeling of the heat and mass transport inside the reactor using CFD/phase-field modeling, and investigation of the synthesis of newer 2D materials offer promising avenues for future research. We hope that our review motivates the use of modeling, simulation, and machine learning tools to better understand and predict the CVD growth of 2D materials.
